# Multimeric immunotherapeutic complexes activating natural killer cells towards HIV-1 cure

**DOI:** 10.1186/s12967-023-04669-4

**Published:** 2023-11-07

**Authors:** Rafaëla Schober, Bianca Brandus, Thessa Laeremans, Gilles Iserentant, Camille Rolin, Géraldine Dessilly, Jacques Zimmer, Michel Moutschen, Joeri L. Aerts, Xavier Dervillez, Carole Seguin-Devaux

**Affiliations:** 1 Department of Infection and Immunity, Luxembourg Institute of Health, 29, Rue Henri Koch, L-4354 Esch-Sur-Alzette, Luxembourg; 2 Neuro-Aging and Viro-Immunotherapy (NAVI) Research Group, Faculty of Pharmacy and Medicine, Vrije Universiteit Brussel, 1090 Brussels, Belgium; 3 AIDS Reference Laboratory, Catholic University of Louvain, Ottignies-Louvain-la-Neuve, Belgium; 4 Department of Infectious Diseases, University of Liège, CHU de Liège, Liège, Belgium

**Keywords:** NK cells, HIV cure, Immunotherapy, IL-15, NKG2A, KIR2DL

## Abstract

**Background:**

Combination antiretroviral therapy (cART) has dramatically extended the life expectancy of people living with HIV-1 and improved their quality of life. There is nevertheless no cure for HIV-1 infection since HIV-1 persists in viral reservoirs of latently infected CD4^+^ T cells. cART does not eradicate HIV-1 reservoirs or restore cytotoxic natural killer (NK) cells which are dramatically reduced by HIV-1 infection, and express the checkpoint inhibitors NKG2A or KIR2DL upregulated after HIV-1 infection. Cytotoxic NK cells expressing the homing receptor CXCR5 were recently described as key subsets controlling viral replication.

**Methods:**

We designed and evaluated the potency of “Natural killer activating Multimeric immunotherapeutic compleXes”, called as NaMiX, combining multimers of the IL-15/IL-15Rα complex with an anti-NKG2A or an anti-KIR single-chain fragment variable (scFv) to kill HIV-1 infected CD4^+^ T cells. The oligomerization domain of the C4 binding protein was used to associate the IL-15/IL-15Rα complex to the scFv of each checkpoint inhibitor as well as to multimerize each entity into a heptamer (α form) or a dimer (β form). Each α or β form was compared in different in vitro models using one-way ANOVA and post-hoc Tukey’s tests before evaluation in humanized NSG tg-huIL-15 mice having functional NK cells.

**Results:**

All NaMiX significantly enhanced the cytolytic activity of NK and CD8^+^ T cells against Raji tumour cells and HIV-1^+^ ACH-2 cells by increasing degranulation, release of granzyme B, perforin and IFN-γ. Targeting NKG2A had a stronger effect than targeting KIR2DL due to higher expression of NKG2A on NK cells. In viral inhibition assays, NaMiX initially increased viral replication of CD4^+^ T cells which was subsequently inhibited by cytotoxic NK cells. Importantly, anti-NKG2A NaMiX enhanced activation, cytotoxicity, IFN-γ production and CXCR5 expression of NK cells from HIV-1 positive individuals. In humanized NSG tg-huIL-15 mice, we confirmed enhanced activation, degranulation, cytotoxicity of NK cells, and killing of HIV-1 infected cells from mice injected with the anti-NKG2A.α NaMiX, as compared to control mice, as well as decreased total HIV-1 DNA in the lung.

**Conclusions:**

NK cell-mediated killing of HIV-1 infected cells by NaMiX represents a promising approach to support HIV-1 cure strategies.

**Supplementary Information:**

The online version contains supplementary material available at 10.1186/s12967-023-04669-4.

## Background

Combined antiretroviral therapy (cART) has transformed HIV-1 infection from a lethal disease into a chronic, manageable infection, considerably improving survival and prevention of transmission. However, HIV-1 persists in reservoirs of latently infected CD4^+^ T cells containing a minor part of integrated replication-competent viral DNA that is transcriptionally silent [1]. Given both the 38 million people living with HIV-1 worldwide and the inability of cART to eradicate the viral reservoir, the HIV epidemic remains one of the greatest health challenges in modern history underlining the importance of continued efforts to find a cure. Two approaches have been investigated in this respect: on the one hand, HIV-1 research has explored several ways directed towards the complete eradication of the reservoir, and on the other hand, various methods aimed at obtaining a long-term remission in the absence of cART, called “functional cure”, have also been attempted. Although this terminology has been largely controverted [2], the concept itself relies on a cART-free durable virologic suppression without clearing the latent reservoirs. The most studied reservoir eradication strategy is the “shock and kill” approach, which relies on the activation of latent reservoir cells by latency reversal agents (LRA) followed by the recognition and elimination of cells harboring the reactivated virus by cytotoxic T lymphocytes (CTL) or natural killer (NK) cells.

NK cell-based immunotherapy was shown to be a promising approach in cancer treatment and is now also more and more proposed as an approach to control HIV-1 infection [3–5]. NK cells are multipotent innate lymphoid cells that have a fundamental function in immune-surveillance against cancer cells and virus infected cells without prior stimulation. They can rapidly recognize stressed cells through a shift in the balance between germline encoded activating and inhibitory NK cell receptors (aNKRs and iNKRs, respectively) and eliminate them through antibody-dependent cellular cytotoxicity (ADCC) mediated by the FcγRIIIA receptor (CD16a) or “natural killing” via exocytosis of lytic granules containing granzymes and perforin. Human NK cells represent 5–15% of circulating lymphocytes and represent an important link to the adaptive immune system through the secretion of cytokines and chemokines and further interactions with dendritic cells. Two main NK cell populations are subdivided by their CD56 expression; the regulatory CD56^bright^ (5–10% of all peripheral blood NK cells), and the cytotoxic CD56^dim^ (90% of all peripheral blood NK cells). While NK cells are one of the first responders during the acute phase of HIV-1 infection and produce a large amount of IFN-γ [6], chronic infection generates abnormal distribution of subpopulations with the expansion of dysfunctional CD56^neg^ cells [7, 8], reduction of the aNKRs expression [9], down-regulation of cytokine production and reduction of stored perforin and granzyme A. cART is only partially able to recover NK cell distribution, cytotoxicity and IFN-γ expression [10, 11], which likely prevents them from clearing the latent reservoir after viral reactivation [12]. B cell follicles (BCF) are the major sanctuary for HIV-1 reservoirs due to the exclusion of immune effector cells and low penetration of cART. Importantly, CXCR5^+^ NK cells accumulated in the lymph nodes of HIV-1 infected patients were inversely correlated with viral load [13].

The ability of interleukin-15 (IL-15) to increase NK and T cell activation, expansion and proliferation has been well-established [14, 15] and novel IL-15 based therapies were extensively tested in oncology [16–19]. IL-15 is a cytokine from the common γ chain family and is predominantly trans-presented by antigen presenting cells (APCs) or cis-presented to target cells by its co-receptor unit α (IL-15Rα) [20]. The concentration of IL-15 must be constantly above a certain threshold to reach an effect on NK cell expansion and activation. However, IL-15 has a very short half-life (2.5 h) making the in vivo administration of a single injection impossible without a high C_max_ and resultant toxicity [21]. Complexing IL-15 to its co-receptor IL-15Rα has proven to increase stability, solubility and even stimulatory activity [22]. IL-15/IL-15Rα based immunomodulatory molecules have shown a high potency to activate and increase cytotoxic activity of NK cells in the case of HIV-1 infection [23–26]. In addition, the IL-15 superagonist ALT-803 (also called N-803), is able to activate latently infected cells, prime resting CD4^+^ T cells for CD8^+^ T cell recognition in vitro and ex vivo*,* and was proposed as a LRA [27]. ALT-803 was also capable of directing effector CD8^+^ T and NK cells to the B cell follicles in SHIV infected macaques [28]. Several clinical trials were started to assess the effect of ALT-803 on the control of HIV-1 infection in humans as a functional cure (NCT04505501), on acute infection (NCT04505501) or as a LRA (NCT04808908).

Blockade of inhibitory receptors such as cytotoxic T-lymphocyte associated protein 4 (CTLA-4) or the Programmed cell death 1 (PD-1)/PD-L1 axis on T lymphocytes is another major strategy applied in cancer immunotherapy [29]. These treatments can induce strong responses, but only in a minority of patients. The activation of NK cells depends on the stimulation balance between aNKRs and iNKRs, making them excellent candidates for immune checkpoint blockade. Potential targets on NK cells are the killer cell immunoglobulin-like receptors 2DL (KIR2DL) or the lectin-like receptor NKG2A. They are inhibitory receptors for human leukocyte antigen class I-C (HLA-C) and α-chain-E (HLA-E), respectively. HLA-C and HLA-E are broadly expressed on healthy tissues to define immune “self”, but HLA-E can be over-expressed on cancer cells or HIV-1 infected T lymphocytes to escape immune recognition [30, 31]. Hence, down-regulation of NKG2A on NK cells increases antitumor activity against HLA-E expressing resistant tumor cells [32] and blocking NKG2A in combination with the PD-1/PD-L1 pathway improves tumor control [33]. Moreover, blocking the KIR2DL1, DL2, and DL3 receptors increased NK cell mediated killing of acute myeloid leukemia cells and activated NK cells from HIV-1 infected viremic and aviremic patients [34].

In this study, we describe the development and the validation of novel therapeutic molecules, called NaMiX, for NK activating Multimeric immunotherapeutic compleXes. The oligomerization domain of C4 binding protein (C4bp) was used to associate the IL-15/IL-15Rα complex with an anti-NKG2A or an anti-KIR single-chain fragment variable (scFv) as well as to multimerize each entity into a heptamer (α form) or a dimer (β form) [35]. The different NaMiX showed increased ability to activate NK cells and to increase their killing against HIV-1-infected and cancer cells in vitro. Preliminary experiments performed in humanized NSG tg-huIL-15 mice confirmed that the NKG2A.IL-15 NaMiX enhanced the cytotoxic capability of NK cells in vivo.

## Methods

### Molecular design of NaMiX

The following cDNA constructs were optimized and synthesized (ProteoGenix SAS, Schiltigheim) for expression of the different molecules: human (hu) IL-15Rα-Sushi (UniProt n°Q13261, aa 31–205)—hu C4bp C-terminal β chain (UniProt n°P20851, aa 137–252)—scFv Z199 (humanized anti-NKG2A, Monalizumab, patent n°US20110052606A1)—5xHis; hu IL-15Rα-Sushi-hu C4bp C-terminal α chain (UniProt n°P04003, aa 540–597)—scFv Z199-5xHis; hu IL-15Rα-Sushi-hu C4bp C-terminal β chain—scFv IPH2102 (anti-KIR2DL1 -2 and -3, Lirilumab, patent n°WO2014/055648Al)—5xHis; hu IL-15Rα-Sushi-hu C4bp C-terminal α chain- scFv IPH2102 (anti-KIR, Lirilumab)—5xHis. All expression cassettes were cloned between BglII and NotI of the multiple cloning site of a bi-cistronic pEF-IRESpac expression vector. The signal peptide from the tumor necrosis factor receptor superfamily member 16/NGFR (UniProt n°P08138) was cloned between EcoRI and BglII. The pcDNA3.1 vector encoding for human IL-15 was synthesized by ProteoGenix SAS (Strasbourg, France).

### Cell culture and antibodies

All molecules were generated from stably transfected human HEK293F cells (ATCC CRL‐1573, RRID: CVCL_0045) cultured in Dulbecco’s modified Eagle’s medium (DMEM) (Gibco, Belgium). Peripheral blood mononuclear cells (PBMCs) isolated from healthy donors (Red Cross Luxembourg), Raji (ATCC CCL-86, RRID: CVCL_0511), a CD20+ Burkett lymphoma derived cell line, and ACH-2 (NIH HIV reagent program ARP-349, RRID: CVCL_0138), an acute lymphoblastic leukemic T-cell line latently infected with HIVLAI cells were cultured in Roswell Park Memorial Institute (RPMI) 1640 Medium (Gibco) with 10 mM HEPES (Gibco), 2 mM l-glutamine, non-essential amino acids (Gibco), 10% heat-inactivated Fetal Bovine Serum (FBS, Life Technologies Europe BV, Belgium), 1 U/ml of penicillin, 1 μg/ml of streptomycin (Pen/Strep, Lonza, Belgium). The NK-92 MI (ATCC CRL-2408, RRID: CVCL_3755) cell line was grown in Alpha-MEM medium (Gibco) with 2 mM l-Glutamine, 12.5% FBS and 12.5% horse serum (Gibco). The myeloid leukemia cell line K562 (ATCC CCL-243, RRID: CVCL_0004) and the HLA-E expressing K562 cells were a kind gift from Thorbald van Hall (Department of Medical Oncology, Leiden University Medical Center), and were cultured in complete RPMI 1640 supplemented with 10% heat-inactivated FBS (Life Technologies Europe BV), 1 U/ml of penicillin, 1 μg/ml of streptomycin and 2 mM of L-glutamine (Lonza). HLA-E expressing K562 cells were sorted and maintained in complete RPMI supplemented with 2 µg/ml blasticidin (Sigma-Aldrich, Belgium). All cells were cultured at 37 ℃ with 5% CO_2_.

The following antibodies were used for ELISA and flow cytometry: IL-15 monoclonal antibody (ct2nu): (16.0157.82, RRID: AB_10596500), PE IL-15 monoclonal antibody: (MA5-23561, RRID: AB_2608837), PE-Cy5 CD14 monoclonal antibody: (15-0149-42, RRID: AB_2573058), PE-Cy5 CD19 monoclonal antibody: (15-0199-42, RRID: AB_10853658) from Thermofisher Scientific. Anti-polyHistidine-Peroxidase antibody: (A7058, RRID: AB_258326), anti-mouse IgG-Peroxidase antibody: (A9044, RRID: AB_258432) from Merck. APC anti-HIS Tag antibody: (362605, RRID: AB_2715818), PE anti-human CD159a antibody: (375104, RRID: AB_2888861), BV605 mouse anti-human CD25: (302632, RRID: AB_11218989), BV711 mouse anti-human CD185 (CXCR5): (356934, RRID: AB_2629526) from Biolegend. BV711 mouse anti-human CD8: (563677, RRID: AB_2744463), BUV495 mouse anti-human CD3: (612940, RRID: AB_2870222), FITC mouse anti-human IFN-y: (552882, RRID: AB_394511), BV421 mouse anti-human CD107a: (562623, RRID: AB_2737685), BUV737 mouse anti-human CD16: (612786, RRID: AB_2833077), BV786 mouse anti-human CD56: (564058, RRID: AB_2738569), BV510 mouse anti-human HLA-DR: (563083, RRID: AB_2737994), PE mouse anti-Stat5: (612567, RRID: AB_399858), PE-CF594 mouse anti-human CD69: (562617, RRID: AB_2737680), AF488 mouse anti-human Ki-67 (558616, RRID: AB_647087) from BD Biosciences. PE CD158a/h (KIR2DL1/DS1) anti-human antibody: (130-099-209, RRID: AB_2660573) from Miltenyi Biotec. APC CD158b1/b2j (KIR2DL2/DL3/DS2) mouse anti-human antibody: A22333, HIV-1 p24 core antigen KiC57: (6604667, RRID: AB_1575989) from Beckman Coulter and APC HIV-1 (p24) human monoclonal antibody 28B7: MM-0289-APC from Medimabs.

### Establishment of stable cell lines for the production of NaMiX

Cells were co-transfected with the bi-cistronic pEF-IRESpac coding for the NaMiX molecules under study and the pcDNA3.1 coding for rhuIL-15. In order to make stable cell lines, HEK293F cells were transfected following the lipofectamine 3000 (ThermoFisher Scientific) manufacturers’ protocol. 24 h prior to transfection, cells were seeded in 2 ml FBS-free OptiMEM (Life Technologies Europe BV) in 6 well plates. Four μg of DNA in a 1:1 ratio was transfected with 5 µl of lipofectamine and 4 μl of reagent. One ml of complete DMEM medium was added 24 h after the transfection. 48 h later, cells were transferred to 10-cm culture dish and cultured in complete DMEM medium supplemented with 5–20 µg/ml of puromycin (InvivoGen) and 100–500 µg/ml geneticine disulfate (G418) (Carl Roth). Clones were expanded in 96 well plates. Supernatants from single-isolated clones were screened using anti-IL-15/anti-His sandwich ELISA as described below.

### Purification of NaMiX

The clones expressing the highest levels of molecules were slowly expanded from 24 well plates to five chamber Corning^®^ cellSTACKs^®^ (Corning) in DMEM complete medium supplemented with the appropriate selection antibiotics. After 24 h the medium was replaced by FBS–free Opti-MEM (Gibco) medium for 48 h. Opti-MEM cultured supernatant was collected, cleared by centrifugation, and filtered using 0.22 µm PVDF 1L vacuum filter units (GE-Healthcare, VWR). Twenty mM final imidazole (Sigma-Aldrich) concentration was added to the Opti-MEM supernatant. Molecules were loaded on a Nickel His-Trap^™^ Excel column (Cytiva) over 48 h on a peristaltic pump at a flow rate of 1 ml/min (GE-Healthcare, VWR). Using a BioLogic DuoFlow 10 system (Bio-Rad Laboratories NV) the column was washed with 20 mM phosphate buffer with 500 mM sodium chloride (NaCl) at pH7.2. In order to increase detachment of all molecules, the column was left overnight in elution buffer (20 mM phosphate buffer with 500 mM of NaCl and 1 M of imidazole at pH7.2). Purified molecules were concentrated on Amicon^®^ Ultra 15, 10KDa MWCO (Millipore-Merck Chemicals NV/SA) and dialyzed against 2 × 3 L of PBS using 10 kDa MWCO Slide-A-lyzer^®^ dialysis cassettes (ThermoFisher Scientific). The final concentration was measured using a NanoDrop^™^ microvolume spectro-photometer (ThermoFisher Scientific).

### Molecular characterization through ELISA

To select the molecules after purification, different ELISA were performed. Purified molecules or mouse anti-human IL-15 antibodies were coated on a MaxiSorp^™^ 96-well flat-bottom *ELISA* plate (ThermoFisher Scientific) for 12 h and for 72 h, respectively (100ng/100 µl PBS/well). All incubations with antibodies were done for 1 h at 4 ℃, washed using 1% PBS (Lonza)/BSA (Carl Roth) and blocked with 5% PBS/BSA. Rabbit anti-His-peroxidase was used for detection of the molecules while the IL-15 detection was done in two steps: first with 100 ng per well of mouse anti-IL-15 and then with 100 ng per well of rabbit anti-mouse IgG conjugated to HRP. Revelation was done with 1 × phosphate citrate buffer (Sigma Aldrich) supplemented with chromogen substrate OPD (ThermoFisher Scientific) and H_2_O_2_ (Sigma Aldrich). The reaction was stopped with H_2_SO_4_. Absorbance was read on the POLARstar Omega (BMG Labtech, Belgium) plate reader at 492 nm and 630nm.

### Flow cytometry analysis

To determine the binding of the molecules on their respective receptors, the purified molecules were incubated for 30 min with either PBMCs from Healthy donors (Red Cross Luxembourg) or cell lines expressing the different receptors. NK-92 MI (ATCC CRL-2408) cells were used for molecules recognizing NKG2A while HEK293F cell lines stably expressing KIR2DL1, KIR2DL2, KIR2DL3 were established to evaluate the molecules recognizing the KIR receptors using pcDNA3.1 vectors (OHu24667C, OHu17046C, OHu55562C) (GenScript). The live cells were stained for NK and CD8^+^ T cell surface markers (anti-CD3, anti-CD8, anti-CD16, anti-CD56, anti-CD14 to gate out monocytes, anti-CD19 to gate out B lymphocytes), with anti-His and anti-IL-15 antibodies and live/dead staining using the LIVE/DEAD^™^ Fixable Near IR Viability kit (L34994, Thermo Fisher Scientific) for all flow cytometry analysis. Acquisition was performed on an LSR Fortessa flow cytometer (BD Bioscience, USA) and analyzed with Kaluza (Beckman Coulter, Brea, California, USA). To evaluate intracellular phosphorylation of STAT5, cells were incubated with the molecules for 1, 10, 20 and 40 min and stained for extracellular markers on ice. Intracellular staining was performed after permeabilisation with Perm buffer III (BD Phosflow^™^) following the manufacturer’s protocol. Cells were acquired by the LSR Fortessa flow cytometer or an ImagestreamX (Amnis Corporation, Seattle, WA, USA).

### Measurement of cytotoxicity and killing against cancer or HIV-1 positive target cells

PBMCs from Healthy donors (Red Cross Luxembourg) or NK cells isolated from these PBMCs by negative selection beads (Miltenyi Biotech) or PBMCs from HIV-1 positive individuals under cART or not were incubated for 24 or 48 h with 3 µg of the molecules or 10 ng rhuIL-15 (StemCell, Belgium) in 1 ml RPMI complete medium. To measure degranulation and production of cytokines, PBMCs were collected and further incubated with anti-CD107a and Raji cells or ACH-2 cells or HLA-E expressing K562 cells at an Effector:Target (E:T) ratio of 10:1 in a 96 V bottom shaped well plate. After 1 h incubation, GolgiStop^™^ and GolgiPlug^™^ (BD Biosciences) were added for another 4 h for intracellular staining of IFN-γ. Finally, cells were washed and stained with Live/Dead staining and NK cell and CD8^+^ T cell surface markers anti-CD3 and anti-CD8 to gate on CD8^+^ T lymphocytes, anti-CD14 and anti-CD19 to exclude monocytes and B lymphocytes, respectively and anti-CD56, anti-CD16, to gate on CD3^−^CD56^+^CD16^+^ NK cells, permeabilized following the Cytoperm/Cytofix protocol (BD Biosciences) and stained with the anti-IFN-γ antibody. For measurement of killing, ACH-2 cells, Raji cells and HLA-E expressing K562 cells were pre-stained with CellTrace^™^ Violet kit (C34557, Invitrogen) following the manufacturer’s protocol. Briefly, target cells were incubated with CellTrace^™^ Violet for 10 min at 37 ℃. Staining was stopped by adding 10 ml complete RPMI and incubating at 37 ℃ for 10 min. PBMCs were collected after 48 h pre-stimulation with the molecules and further incubated with the pre-stained target cells at an E.T ratio of 10:1 in a 96 V bottom shaped well plate for 5 h. In the case of ACH-2 cells, HIV-1 positive or negative sera (diluted 1:1000) were added to evaluate ADCC. After incubation, all cells were L/D stained and acquisition was performed on the LSR Fortessa flow cytometer (BD Biosciences) and analyzed with Kaluza (Beckman Coulter). Cytokine secretion in the supernatant was evaluated by ELISA using MAX Deluxe Set Human IFN-γ (BioLegend), Human Perforin ELISA^BASIC^ kit (HRP) (MabTech), and Human Granzyme B ELISA^BASIC^ kit (HRP) (MabTech) following the manufacturer’s instructions. To investigate NaMiX-mediated activation of PBMCs and further killing of ACH-2 cells, PBMCs from healthy donors (Red Cross Luxembourg) were prestimulated by NaMiX or controls for 48 h. CD25 and CD69 expression of NK and CD4^+^ T cells were measured by flow cytometry after being incubated with ACH-2 cells for 6 h. HIV-1 replication via the release of HIV-1 mRNA in the supernatant was quantified by ddPCR as previously described [36] after 24 h of co-culture.

### Viral inhibition assay

PBMCs from HIV-1 infected patients on cART were thawed and cultured for 24 h in complete RPMI medium. CD4^+^ T cells were isolated from the rested PBMC’s using positive selection beads (Miltenyi Biotech) according to the manufacturer’s instructions. After isolation, CD4^+^ T cells were activated for 24 h in RPMI medium with IL-2 (500 IU/ml) and Phytohemagglutinin (PHA) at 5 µg/ml. Natural killer cells were isolated using negative selection beads (Miltenyi Biotech) from the unlabeled cell fraction of the previous CD4^+^ T cell selection. After cell isolation, purity of CD4^+^ T cells and NK cells were confirmed by flow cytometry. Natural killer cells were cultured for 24 h at a density of 2.10^6^ cells/ml in RPMI medium with and without molecules. Activated CD4^+^ T cells were washed twice with RPMI medium and infected with 50 ng/ml HIV III-B lab strain (NIH HIV reagent program ARP-2222) using spinoculation at 1200 *g* for 2 h at 25 ℃. Infected CD4^+^ T cells and NK cells were washed twice with PBS and resuspended in RPMI medium with 50 IU/ml of IL-2 at an E:T ratio of 1:1 for two or 5 days. Levels of p24 antigen in supernatant were quantified by p24 ELISA (PerkinElmer) according to the manufacturer’s instructions. Levels of intracellular p24 were quantified using flow cytometry. Cells were stained with anti-CD3, anti-CD4, anti-CD56 and Live/Dead (Thermo Fisher Scientific). Double p24 intracellular staining was performed after permeabilization using the BD Cytofix/Cytoperm kit (BD, Biosciences) following manufacturer’s instructions with the following p24 antibodies: HIV-1 p24 clone 28B7-APC (Medimabs) and HIV-1 p24 clone KC57-PE (Beckman Coulter). Acquisition was performed on the LSR Fortessa flow cytometer (BD Biosciences, USA). Viral mRNA was measured in the supernatant as previously described [36].

### NSG mice immune reconstitution, HIV-1 infection and treatment with cART

NSG (NOD/LtSz-scid/IL2Rγnull) (RRID: IMSR_JAX:005557, Charles River Laboratory, France) and NSG tg-huIL-15 (RRID: IMSR_JAX:030890, Jackson Laboratory, France) were maintained and bred in a specific pathogen free animal facility of the Luxembourg Institute of Health. All experiments on animals were performed with the authorizations from the animal welfare committee of the Luxembourg Institute of Health and the Ministry of Veterinary and Agriculture of Luxembourg (protocol numbers: DII-2018-21 and DII-2019-02), and complied with the national legislation and guidelines for animal experimentation in Luxembourg. Mice were humanized as we described previously [36] with CD34^+^ hematopoietic stem cells isolated from human cord blood (CB) using a magnetic activated cell sorting CD34^+^ progenitor cell isolation kit (Stem Cell Technologies, Belgium). Cord blood was provided by the Cord Blood Bank Central Hospital University (Liège, Belgium). Animals that had over 20% of circulating human CD45^+^ cells were infected by two intraperitoneal (IP) injections of the HIV-1 laboratory adapted strain JRCSF (ARP-2708 HIV-1 Strain JR-CSF Infectious Molecular Clone pYK-JRCSF, NIH HIV reagent program, 10.000 TCID50) within 24 h. Infection was monitored by viral load measurement every one or 2 weeks on plasma from blood samples collected by submandibular bleeding. cART treatment was initiated 4 weeks post-infection and continued for a total of 6 weeks. Single tablets of dolutegravir/abacavir/lamivudine (Triumeq^®^, ViiV Healthcare) were crushed and dissolved in Sucralose MediDrop^®^ (Clear H_2_O) at the therapeutic concentration of 3.40 mg/ml. Drinking solution was refreshed twice per week. Therapeutic molecules or PBS were injected 10 or 3 days prior to cART interruption by IV injection at a concentration of 0.2 mg/kg. Viral load was determined as described previously by digital droplet PCR [36]. Four mice per group were sacrificed at the end of the treatment and four mice per treatment were sacrificed 6 weeks after treatment interruption. Blood, spleen, lungs, bone marrow were collected and processed immediately for cell staining, phenotyping, degranulation, and cytotoxic activity as described above. Human CD45^+^ cells were purified using positive selection beads (Miltenyi Biotech, Germany) and total HIV-1 DNA was measured by PCR in the bone marrow and the lung by ddPCR as previously described [36].

### Statistical analysis

Statistical analysis was performed using GRAPHPAD PRISM software. Data were expressed as the mean value ± SD. For all in vitro experiments, multiple groups receiving the different molecules were compared using one-way ANOVA and post-hoc Tukey’s tests. For studies in mice, an appropriate sample size (n = 5) was calculated during the study design to obtain groups with a difference of humanization of 10% by taking into account a common standard deviation of 5% using a bilateral Student’s T test based on a 95% confidence level and homogeneous viral load [36].The level of humanization and viremia was randomized between the groups. The two groups were compared using unpaired Student’s T test. A p < 0.05 was considered to be significant.

## Results

### Molecular characterization

**Fig. 1:**
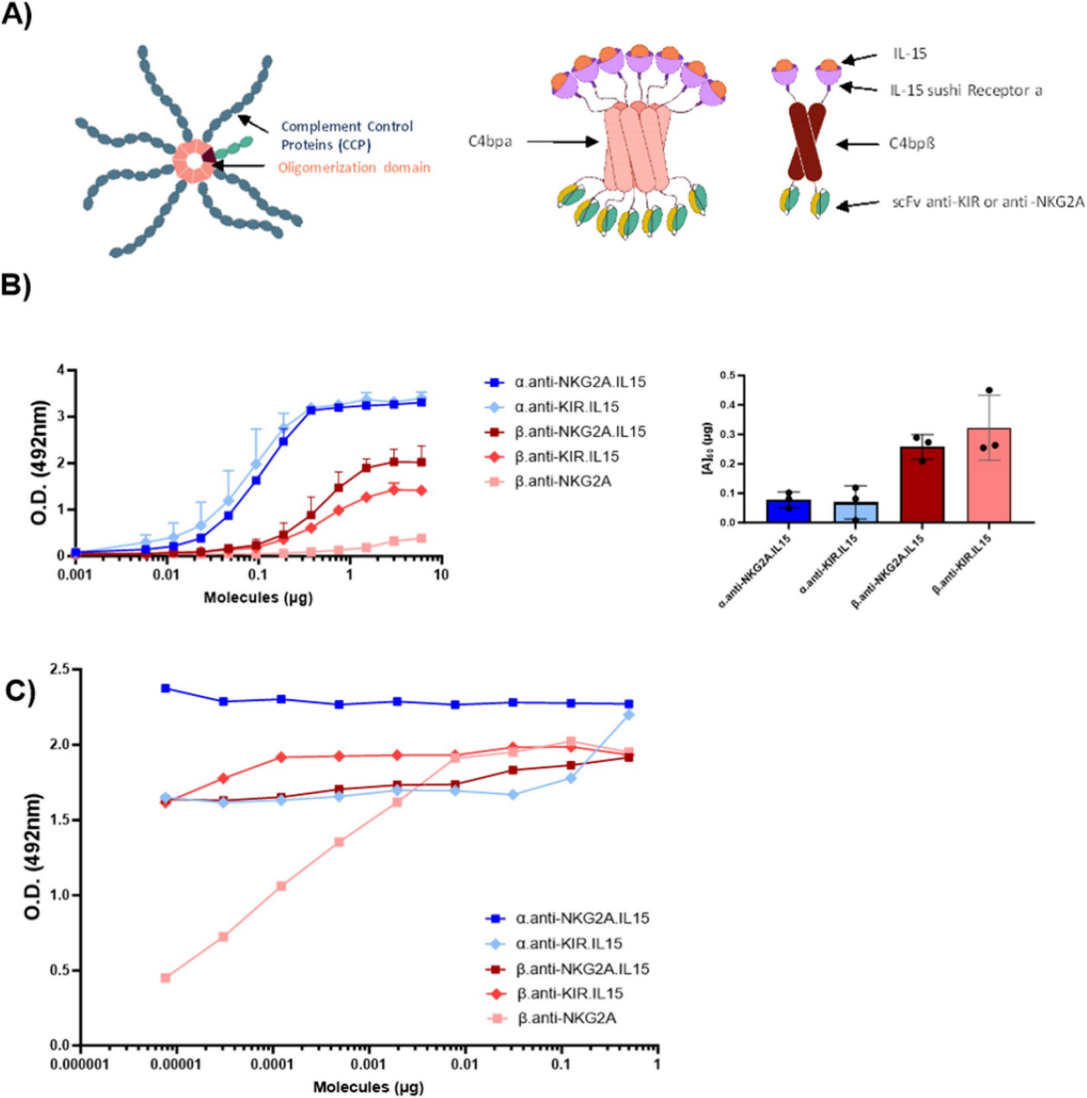
Demonstration of the huIL-15/IL-15Rα complex formation on purified NaMiX. **A** Schematic representation of the multimeric molecules. The C4bp alpha forms multimerize in heptamers while the C4bp beta forms multimerize in dimers. The anti-NKG2A or anti-KIR scFvs are located in the C-terminal part of the C4bp moiety while IL15Rα is located at the N-terminus. The plasmids coding for the molecules are co-transfected with a plasmid encoding for recombinant human IL-15. **B** Polystyrene MaxiSorp^™^ plates were coated with a mouse anti-human IL-15 mAb, incubated with a two-fold serial dilution of molecules (6 μg to 0.001 μg) and binding of the molecules was detected with a mouse anti-HIS pAb HRP-conjugated. Figure **B** represent triplicates of three independent experiments; data are expressed as the mean value ± SD. The right panel represents the concentration of NaMiX needed to half saturate the ELISA (on the left panel). **C** Saturating concentrations of molecules were coated on Polystyrene MaxiSorp^™^ plates (6 μg for β forms and 3 μg for α forms). A serial dilution of rhuIL-15 was added to each molecule. The binding of IL-15 was detected with a mouse anti-human IL-15 mAb followed by a goat anti-Mouse IgG HRP-conjugated polyclonal Ab. Figure **C** shows the data of one representative experiment. NaMiX engrafted with IL-15Rα/IL-15 using C4bpα or C4bpβ and expressing the anti-NKG2A or the anti-KIR scFv are so called: α.anti-NKG2A.IL-15, β.anti-NKG2A.IL-15, α.anti-KIR.IL-15 and β.anti-KIR.IL-15, respectively while the control NaMiX without IL-15Rα/IL-15 expressing the anti-NKG2A scFv is termed β.anti-NKG2A

C4bp is a protein complex composed of seven identical α chains and a single β chain, which inhibits the lectin and classical pathways of the complement system. Each subunit is composed of several complement control proteins (CCPs) and an oligomerizing C-terminal domain. The inactive oligomerizing entities of C4bpα and C4bpβ have the unique ability of forming heptamers and dimers, respectively, by forming disulfide bounds (Fig. 1A). We previously reported that the fusion of effector or target entities into this domain does not affect its oligomerizing capacity and can be used to increase its half-life [35] and functionality of chosen proteins [37]. To express IL-15 heptamers (α molecules) or dimers (β molecules) at the surface of NK cells and generate NaMiX, we grafted the extracellular sushi domain of IL-15Rα on the N-terminus of the C4bp oligomerization domain and the scFvs of anti-NKG2A or anti-KIR on the C-terminus. All molecule-encoding sequences were cloned into pEF-IRES-pac expression vectors containing a histidine (His) tag and were co-transfected into HEK293F cells transfected with a rhuIL-15 coding pcDNA3.1 to produce α.anti-NKG2A.IL-15, β.anti-NKG2A.IL-15, α.anti-KIR.IL-15 and β.anti-KIR.IL-15 NaMiX. Control molecules without IL-15 were also generated for each anti-NKG2A or anti-KIR NaMiX. However, the only control molecule that was produced in sufficient amount without co-transfection with rhuIL-15, was β.anti-NKG2A, so the controls α.anti-NKG2A, α.anti-KIR and β.anti-KIR could not be considered.

We first assessed the binding efficiency of IL-15 to its co-receptor IL-15Rα, using an anti-IL-15/anti-His sandwich ELISA on increasing concentrations of the NaMiX (Fig. 1B). The β.anti-NKG2A.IL-15 and β.anti-KIR.IL-15 forms required 0.26 and 0.32 μg of each NaMiX, respectively, to reach a 50% saturation, while the α.anti-NKG2A.IL-15 and α.anti-KIR.IL-15 forms only required 0.07 and 0.06 μg of each NaMiX, respectively. Full saturation was reached at 0.375 μg for the α form and at 1.5 μg for the β forms. Indeed the α forms of the molecules have seven His tags and IL-15 entities whereas the β forms only have two entities of each, explaining the fourfold higher concentration needed to reach a plateau. Next, we wanted to determine if all IL-15Rα sites were saturated with IL-15 and whether external rhuIL-15 could still bind to NaMiX. When adding external rhuIL-15 to saturating concentrations of β.anti-NKG2A, it required 7.8 ng of rhuIL-15 to saturate 6 μg of the molecule indicating that 3 μg of the β.anti-NKG2A.IL-15 NaMiX used in this study is approximatively equivalent to 3.9 ng of rhuIL-15. As shown in Fig. 1C, all sites of α.anti-NKG2A.IL-15 were saturated with IL-15 while α.anti-KIR.IL-15 required an additional 122 pg rhuIL-15 and β.anti-NKG2A.IL-15, and β.anti-KIR.IL-15 required an additional 0.5 μg rhuIL-15 to saturate all sites of the molecules.

### NaMiX bind to their respective receptors NKG2A and KIR2DL1/2DL2/2DL3

We first confirmed by flow cytometry that all NaMiX bind to their respective receptors using KIR2DL1, 2DL2, 2DL3- expressing HEK293F cells and on NKG2A-expressing NK-92MI cells (Additional file 1: Fig. S1). Both α.anti-KIR.IL-15 and β.anti-KIR.IL-15 were found to bind to all target KIR2DL receptors expressed on stable cell lines (Additional file 1: Fig. S1A). Similarly, α.anti-NKG2A.IL-15, β.anti-NKG2A.IL-15 and the β.anti-NKG2A control molecule, all bound to the NKG2A expressing NK-92MI cell line (Additional file 1: Fig. S1B). Since the NKG2A and KIR receptors are heterogeneously expressed on human NK and CD8^+^ T cells, we measured their expression on PBMCs from healthy donors (Additional file 1: Fig. S1C) and evaluated the subsequent binding of NaMiX on these cells. NKG2A was expressed on more than 50% of the CD3^−^CD56^+^CD16^+^ NK cells whereas the total expression of all KIRs did not exceed 35% of PBMC-derived NK cells. As shown by the His staining, the α.anti-NKG2A.IL-15 NaMiX bound to 83.7% ± 4.041% of all NK cells while the β.anti-NKG2A.IL-15 form only bound to 20.7% ± 11.21% of NK cells (Additional file 1: Fig. S1D), in agreement with the respective number of anti-NKG2A scFv valences of the α heptamer and the β dimer. When looking at the IL-15 positive NK cells, although the reduced binding between the β and α form of the NKG2A NaMiX was significant (p = 0.0006), α.anti-KIR.IL-15 did not show a higher signal than β.anti-KIR.IL-15 (p = 0.998) suggesting a lower IL-15 amount on α.anti-KIR.IL-15 than α.anti-NKG2A.IL-15, in accordance with data from Fig. 1C.

### NaMiX induce higher STAT5 phosphorylation in NK and CD8^+^. T cells than recombinant human IL-15

Upon binding to IL-15, the cytokine receptor complex recruits the tyrosine kinases JAK1 and JAK3 to phosphorylate STAT5 (pSTAT5) and induce signaling pathways leading to cell survival [38, 39]. We investigated the JAK/STAT5 phosphorylation pathway on PBMCs by intracellular staining using flow cytometry and multispectral imaging cytometry. We first confirmed the intracellular STAT5 phosphorylation by flow cytometry and imaging flow cytometry (Additional File 1: Fig. S2) and observed that all molecules containing IL-15 induce pSTAT5 signal on NK cells and CD8^+^ T cells, whereas β.anti-NKG2A without IL-15 and medium control showed no signal. We further observed an overall strong increase in pSTAT5 positive NK and CD8^+^ T cells early after incubation with α.anti-NKG2A.IL-15 compared to recombinant human IL-15 (rhuIL-15) alone and β.anti-NKG2A.IL-15 (Additional File 1: Fig. S2A). Finally, STAT5 was less phosphorylated by anti-KIR.IL-15 NaMiX than by α.anti-NKG2A.IL-15 in NK cells and the β.anti-NKG2A control molecule did not induce any STAT5 phosphorylation (Additional File 1: Fig. S2B).

### NaMiX significantly increase NK cell degranulation and cytotoxic activity of NK cells

**Fig. 2:**
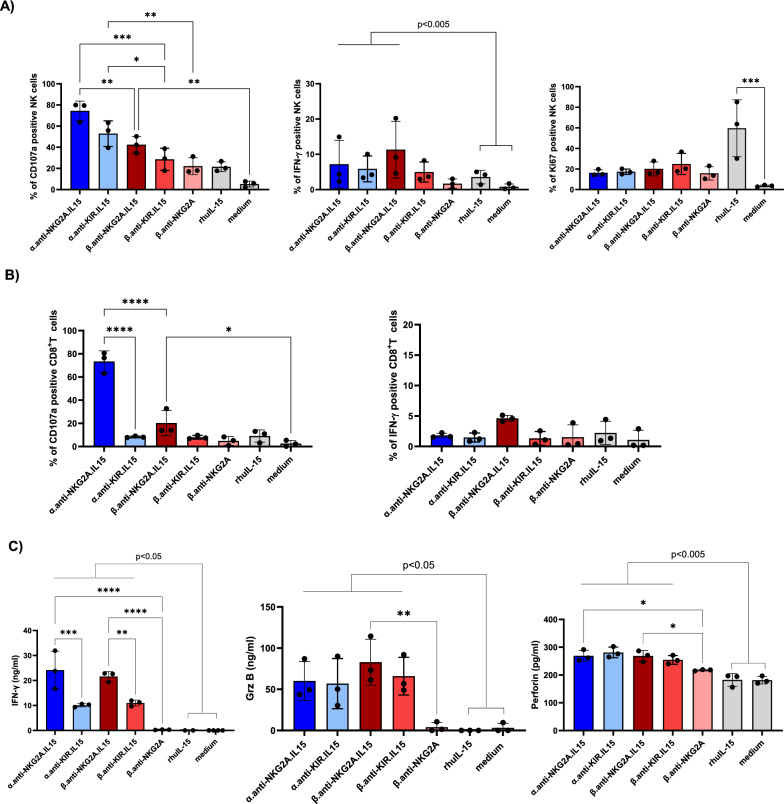
NaMiX increased cytokine production and cytolytic activity of PBMCs but did not increase Ki-67 expression. Human PBMCs were pre-incubated for 24 h (IFN-γ expression) or 48 h (CD107a expression and Ki67 expression) with NaMiX. Cells were further stained for extracellular markers to identify CD3^−^CD56^+^CD16^+^ NK cells (**A**) and CD3^+^CD8^+^. T cells (**B**) using anti-CD3, CD8, CD14, CD16, CD19 and CD56 antibodies. **C** After 48 h incubation, the supernatant was tested for the presence of IFN-γ (left panel), granzyme B (middle panel) and perforin (right panel) by ELISA. The figures represent three independent experiments with three different healthy donors. NaMiX engrafted with IL-15Rα/IL-15 using C4bpα or C4bpβ and expressing the anti-NKG2A or the anti-KIR scFv are so called: α.anti-NKG2A.IL-15, β.anti-NKG2A.IL-15, α.anti-KIR.IL-15 and β.anti-KIR.IL-15, respectively while the control NaMiX without IL-15Rα/IL-15 expressing the anti-NKG2A scFv is termed β.anti-NKG2A, the recombinant human IL-15 as rhIL-15, and the control condition without any molecules as medium. Data were expressed as the mean value ± SD. Statistical analysis was performed using a one-way ANOVA and post-hoc Tukey test (*p < 0.05, **p < 0.005, ***p < 0.001, ****p < 0.0001)

We first evaluated the effect of NaMiX on cytokine expression and cytolytic function of PBMCs as the primary control of all further experiments to verify their specific mode of action. Using flow cytometry, we observed that the NaMiX anti-NKG2A.IL-15, β.anti-NKG2A.IL-15 and α.anti-KIR.IL-15 induce higher expression of the degranulation marker CD107a on NK cells after 48 h of incubation (Fig. 2A) and IFN-γ after 24 h of incubation (Fig. 2B) than rhuIL-15 (p < 0.005). Only anti-NKG2A.IL-15 and β.anti-NKG2A.IL-15 stimulated CD107a expression on CD8^+^ T cells but did not induce significant levels of IFN-γ expression. We did not find any effect of NaMix when measuring the number of living cells in PBMCs 24 h or 48 h after stimulation as compared to the medium control (data not shown) indicating that NaMix has no cytotoxic effect on PBMCs.

Since IL-15 stimulates NK cell proliferation, we further measured the expression of Ki-67 after 48 h of stimulation with NaMiX and observed that the expression of the proliferation marker was not significantly enhanced as compared to rhuIL-15 (Fig. 2C). Regarding IFN-γ secretion after 48 h stimulation (Fig. 2D), all molecules except β.anti-NKG2A resulted in a significantly increased secretion as compared to medium control or treatment with rhuIL-15. However, α.anti-NKG2A.IL-15 and β.anti-NKG2A.IL-15 had a stronger effect than α.anti-KIR.IL-15 and β.anti-KIR.IL-15 on IFN-γ secretion, respectively (p < 0.01) (Fig. 2C). Furthermore, β.anti-NKG2A NaMiX without IL-15 did not stimulate IFN-γ secretion suggesting that blocking of the target receptors with the anti-NKG2A scFv had no impact. Regarding granzyme B and perforin, their concentrations were significantly increased by all NaMiX compared to medium control and rhuIL-15, excepted for the control β.anti-NKG2A for granzyme B, similarly to rhuIL-15. Taken together, these data indicate that PBMCs were stimulated by NaMiX to degranulate and produce cytokines, even in the absence of target cells, but were not induced to proliferate.

**Fig. 3:**
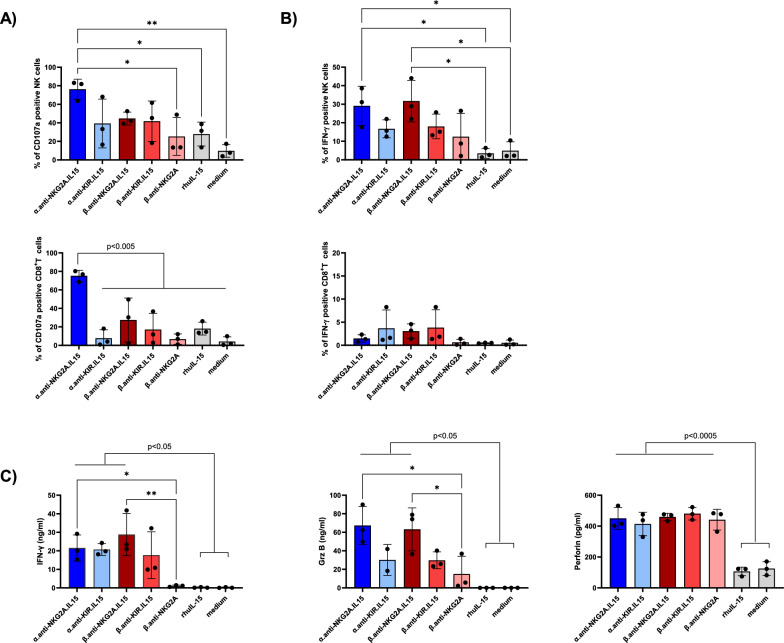
NaMiX increased degranulation and IFN-γ secretion of NK cells against resistant Raji cells. Human PBMCs were pre-incubated for 24 h (for IFN-γ expression) or 48 h (for CD107a expression) with NaMiX and stimulated with Raji cells for 5 h in the presence of anti-CD107a mAb. **A** Cells were further stained for extracellular markers to identify CD107a expression on CD3^−^CD56^+^CD16^+^ NK cells (upper panel) and CD3^+^CD8^+^ T cells (lower panel) using anti-CD3, CD8, CD14, CD16, CD19 and CD56 antibodies. **B** Cells were also permeabilized and further stained for IFN-γ (NK cells on upper panel and CD8^+^. T cells on lower panel). **C** After 48h incubation, supernatant was collected and analysed by ELISA for IFN-γ (left panel), granzyme B (middle panel) and perforin secretion (right panel). NaMiX engrafted with IL-15Rα/IL-15 using C4bpα or C4bpβ and expressing the anti-NKG2A or the anti-KIR scFv are so called: α.anti-NKG2A.IL-15, β.anti-NKG2A.IL-15, α.anti-KIR.IL-15 and β.anti-KIR.IL-15, respectively while the control NaMiX without IL-15Rα/IL-15 expressing the anti-NKG2A scFv is termed β.anti-NKG2A, the recombinant human IL-15 as rhIL-15, and the control condition without any molecules as medium. Figure **A** to **C** represent three independent experiments. Data were expressed as the mean value ± SD. Statistical analysis was performed using a one-way ANOVA and post-hoc Tukey test (*p < 0.05, **p < 0.005)

We next evaluated the capacity of NaMiX to induce NK cell degranulation and cytotoxicity against Raji tumor cells using PBMCs stimulated with the different molecules for 24 and 48 h. Raji is a B-lymphoma cell line constitutively expressing CD20 and overexpressing multiple HLA class-I molecules, making it resistant to NK cell recognition and killing in the absence of additional stimuli. Pre-stimulation of PBMCs for 48h with α.anti-NKG2A.IL-15 significantly increased surface expression of the degranulation marker CD107a on NK (mean 76.25% ± 10.89%) and CD8^+^ T cells (mean 75.22% ± 6.07%) compared to rhuIL-15 (p = 0.0405 and p = 0.0010, respectively) and medium control (p = 0.0036; p = 0.0001, respectively) against Raji cells whereas the anti-KIR.IL15 molecules had no significant effect (Fig. 3A). Furthermore, anti-KIR.IL-15 did not affect CD107a expression on CD8^+^ T cells despite a slightly higher KIR2DL1/DS1 receptor expression compared to NKG2A receptors as shown in Additional File 1: Fig. S1C. Importantly, 24 h stimulation with α.anti-NKG2A.IL-15 and β.anti-NKG2A.IL-15 molecules improved the ability of NK cells to express IFN-γ (mean 29.09% ± 10.61% and mean 31.68% ± 11.23%, respectively) against Raji target cells compared to rhuIL-15 stimulation alone (p = 0.028; p = 0.014, respectively) (Fig. 3B), which was not observed in CD8^+^ T cells (Fig. 3B). Moreover, β.anti-NKG2A stimulation showed no increase in IFN-γ secretion compared to medium control (Fig. 3B, C) indicating that the effect of the molecules on IFN-γ expression and secretion originates from IL-15 stimulation rather than the blocking of NKG2A. The α.anti-NKG2A.IL-15 was superior to increase CD107a, only in CD8^+^ T cells, after 48 h of stimulation, as compared to the other molecules, but this was not the case in NK cells, and was not in accordance with the expression/secretion of IFN-γ, granzyme B and perforin. When looking at the granzyme B secretion, we only observed a significant increase when PBMCs were stimulated with the NKG2A NaMiX (both α- and β-based NaMiX) compared to rhuIL-15 and medium control, whereas perforin was increased with all molecules except with the β.anti-NKG2A control (Fig. 3C). Overall, higher NK cells degranulation, production of cytokines and cytolytic function by NaMix was observed in presence of target cells. In summary, these results showed that both α.anti-NKG2A.IL-15 and β.anti-NKG2A.IL-15 have a strong potency on increasing cytotoxic activity of NK and CD8^+^ T cells against resistant cancer cells.

### NaMiX significantly increase NK cell degranulation and cytotoxic activity against ACH-2 cells

**Fig. 4:**
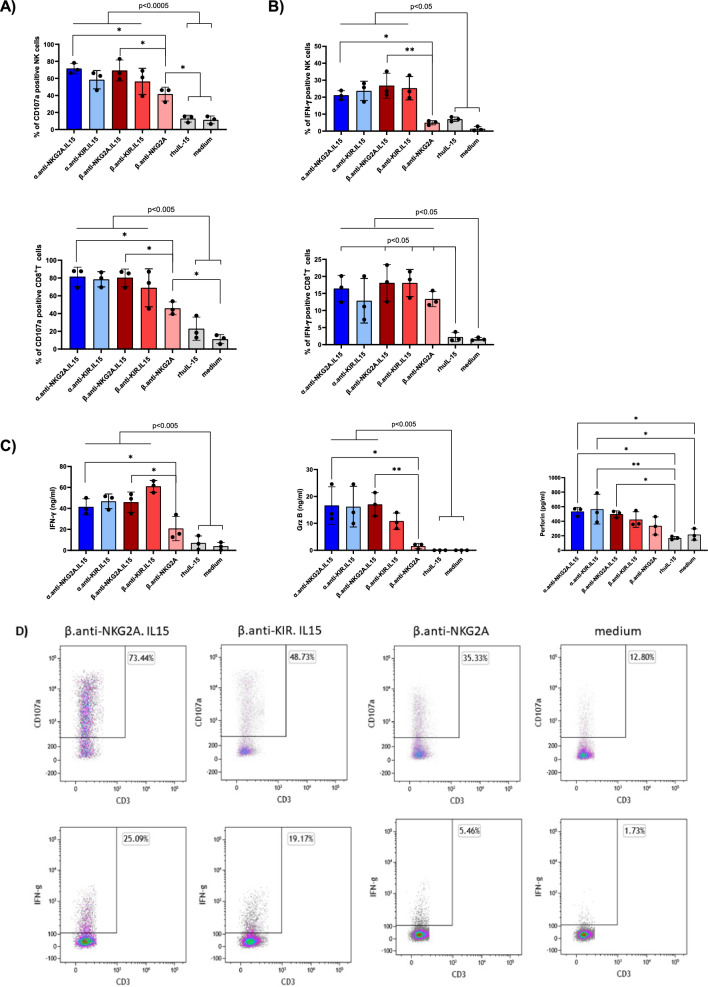
NaMiX increased degranulation and IFN-γ secretion of PBMCs against ACH-2 cells. Human PBMCs were pre-incubated for 24 h (for IFN-γ expression) or 48 h (for CD107a expression) with the NaMiX and stimulated with Raji cells for 5 h in the presence of anti-CD107a mAb.** A** Cells were further stained for extracellular markers to identify CD107a expression on CD3^−^CD56^+^CD16^+^ NK cells (upper panel) and CD3^+^CD8^+^ T cells (lower panel) using anti-CD3, CD8, CD14, CD16, CD19 and CD56 antibodies. **B** Cells were also permeabilized and further stained for IFN-γ (NK cells on upper panel and CD8^+^ T cells on lower panel). **C** After 48 h incubation, supernatant was collected and analyzed by ELISA for IFN-γ (left panel), granzyme B (middle panel) and perforin secretion (right panel). Figure **A** to **C** represent three independent experiments. Data were expressed as the mean value ± SD. Statistical analysis was performed using a one-way ANOVA and post-hoc Tukey test (*p < 0.05, **p < 0.005). **D** Representative expression of CD107a and INF- γ on NK cells pre-activated by NaMiX for 48 h and 24 h, respectively, for one donor included in panel A and B. NaMiX engrafted with IL-15Rα/IL-15 using C4bpα or C4bpβ and expressing the anti-NKG2A or the anti-KIR scFv are so called: α.anti-NKG2A.IL-15, β.anti-NKG2A.IL-15, α.anti-KIR.IL-15 and β.anti-KIR.IL-15, respectively while the control NaMiX without IL-15Rα/IL-15 expressing the anti-NKG2A scFv is termed β.anti-NKG2A, the recombinant human IL-15 as rhIL-15, and the control condition without any molecules as medium

We further tested the effect of the molecules on NK and CD8^+^ T cell degranulation and cytotoxicity against ACH-2 cells, an acute lymphoblastic leukemia T-cell line containing a single integrated copy of HIV-1 per cell. Viral expression in ACH-2 cells induced by PHA/PMA mediated cell activation [40] was first confirmed in the latently infected cell line which resulted in high intracellular p24 expression (data not shown). Stimulation with NaMiX for 48 h had a greater effect on CD107a surface expression of NK cells and CD8^+^ T cells against HIV-1 positive cells than rhuIL-15 alone (Fig. 4A). Interestingly, β.anti-NKG2A without IL-15 (mean 60.7% ± 6.47%) had a similar effect than the other molecules, suggesting that blocking the NKG2A receptor in the context of HIV-1 infection had a more robust effect on degranulation as compared to Raji cells. The secretion of IFN-γ by NK cells was similarly increased by all IL-15 NaMiX (except for β.anti-NKG2A) compared to rhuIL-15 and medium control. Surprisingly, all molecules, even β.anti-NKG2A without IL-15 also increased IFN-γ secretion by CD8^+^ T cells (Fig. 4B). This indicates that blocking the NKG2A pathway is sufficient to enhance IFN-γ production by CD8^+^ T cells but not by NK cells. These data were further confirmed by measuring IFN-γ, granzyme B and perforin secretion in the supernatant (Fig. 4C). All NaMiX showed enhanced IFN-γ, granzyme B and perforin secretion into the supernatant except the β.anti-NKG2A control compared to rhuIL-15 and medium alone (Fig. 4C). To evaluate the potential involvement of ADCC, we also performed the experiment in the presence of sera from HIV-1 positive and negative donors, respectively, and observed no differences in the degranulation capacity or IFN-γ expression of NK and CD8^+^ T cells between both conditions (data not shown). These data suggest that the increase in degranulation and cytokine release by NaMiX is independent of ADCC and relies only on direct cytolytic activity.

### NaMiX significantly enhance NK cell killing of Raji and ACH-2 cells

**Fig. 5:**
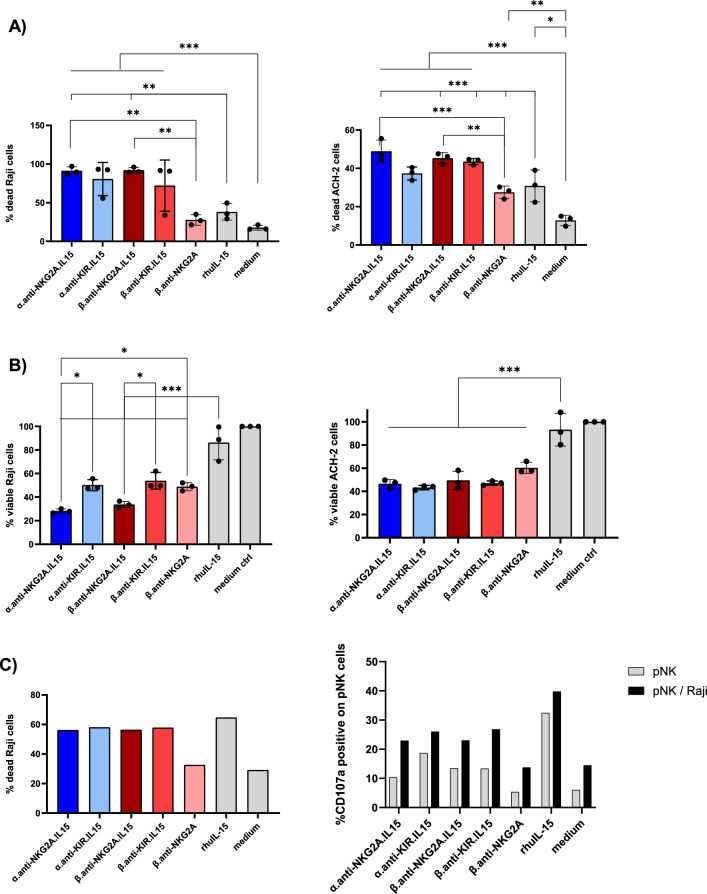
NaMiX enhanced the cytotoxic killing activity of NK and CD8^+^ T cells against Raji and ACH-2 cells. Human PBMCs were pre-incubated for 48 h with the different NaMiX. **A** Stimulated PBMCs were incubated with Raji (left panel) or ACH-2 (right panel) cells pre-stained with CellTrace Violet for 5 h. Cells were further stained using a Live/Dead marker and analyzed by flow cytometry. **B** Stimulated PBMCs were incubated with Raji (left panel) or ACH-2 (right panel) cells for 5 h and the CCK8 reagent was added after 2 h. Absorbance was read at 450 nm. The figures represent three independent experiments with three different donors. Data were expressed as the mean value ± SD. Statistical analysis was performed using a one-way ANOVA and post-hoc Tukey test (*p < 0.05, **p < 0.005, ***p < 0.001). **C** Primary NK cells were pre-stimulated for 24 h with the different NaMiX and incubated or not with Raji cells pre-stained with CellTrace Violet for 5 h in the presence of CD107a (right panel). Cells were further stained using a Live/Dead marker and analyzed by flow cytometry (left panel). Both panels show the data of one representative donor due to the high variability of the response in primary NK cells. NaMiX engrafted with IL-15Rα/IL-15 using C4bpα or C4bpβ and expressing the anti-NKG2A or the anti-KIR scFv are so called: α.anti-NKG2A.IL-15, β.anti-NKG2A.IL-15, α.anti-KIR.IL-15 and β.anti-KIR.IL-15, respectively while the control NaMiX without IL-15Rα/IL-15 expressing the anti-NKG2A scFv is termed β.anti-NKG2A, the recombinant human IL-15 as rhIL-15, and the control condition without any molecules as medium

We next evaluated the killing capacity of NK and CD8^+^ T cells pre-stimulated for 48 h with NaMiX against the targets Raji or ACH-2. In Fig. 5A, PBMCs pre-incubated with all IL-15-containing NaMiX showed a strong killing activity ranging from 70 to 90% against Raji cells when measuring Live/Dead cells by flow cytometry while rhuIL-15 alone, β.anti-NKG2A and the medium control showed a killing activity of 38% (± 10.51%), 28% (± 6.9%) and 18% (± 3.16%), respectively. Furthermore, β.anti-NKG2A.IL-15 stimulated PBMCs killed 92% (± 3.62%) of Raji cells as compared to 28% (± 6.91%) for β.anti-NKG2A, emphasizing the requirement of IL-15 for an optimal cytotoxicity against resistant cancer cells. However, when measuring dehydrogenase activity as a marker of cell viability, (Fig. 5B), α.anti-NKG2A.IL-15 and β.anti-NKG2A.IL-15 stimulation showed a significantly higher killing activity compared to either α or β.anti-KIR.IL-15, respectively (p = 0.036 and p = 0.044, respectively).

When using ACH-2 cells, all NaMiX significantly increased the capacity of PBMCs to kill target cells to a similar level compared to rhuIL-15 alone (Fig. 5B). When measuring the lactate dehydrogenase (LDH) activity in the supernatant, no differences were observed between the NaMiX with a mean of 40% of viable cells (Fig. 5B). We also evaluated whether ADCC could be implicated in the killing of the target ACH-2 cells by adding sera from HIV-1 positive or negative donors, respectively, and did not observe any variation in the killing capacity (data not shown).

To validate the specific effect of NaMix on NK cells, we isolated primary NK cells from PBMCs (Red Cross Luxembourg) by microbeads negative selection and stimulated them with the different NaMiX for 24 h (Fig. 5C). We did not find any cytotoxic effect of NaMix when measuring the number of living NK cells: A mean of around 70% of NK cells were still alive when stimulated with NaMix for 24 h as compared to a mean of around 60% in the medium control (data not shown). All NaMiX with IL-15 induced the killing of almost 55% of Raji cells when NK cells were further incubated with Raji cells for 5 h, similarly to rhuIL-15 (Fig. 5C). Upon stimulation with NaMix, increased CD107a expression was detected in the presence of Raji cells as compared to NK cells alone (Fig. 5C), and correlated with the cytotoxic effects of NaMiX. Altogether, these data confirmed that the IL-15 NaMiX increase the capacity of PBMCs and NK cells to kill cancer cells and HIV-1 positive target cell lines.

### IL-15 stimulation and blocking of NKG2A-HLA-E interaction are required for NK cell activation

Since we observed an increased activation and cytotoxicity of NK and CD8^+^ T cells stimulated with β.anti-NKG2A without IL-15 when co-incubated with ACH-2 cells, we wanted to deeper evaluate whether HLA-E blocking is involved in the effect of the anti-NKG2A NaMiX. We therefore used K562 cells, which are devoid of all MHC molecules, to exclusively express HLA-E. Thus, PBMCs were incubated together with the anti-NKG2A NaMiX and K562 cells with or without HLA-E expression for 5 h. We observed that blocking HLA-E with β.anti-NKG2A alone was insufficient to establish CD107a surface appearance and IFN-γ expression by NK cells compared to medium control (Additional File 1: Fig. S3A). Both α and β.anti-NKG2A.IL-15 significantly increased CD107a expression as compared to β.anti-NKG2A alone (p < 0.005) or rhuIL-15 (p < 0.001). Furthermore, only the α.anti-NKG2A.IL-15 was able to significantly increase IFN-γ expression of NK cells compared to medium control and rhuIL-15 (p = 0.0481 and p = 0.0012, respectively) (Additional File 1: Fig. S3B). Interestingly, even though α.anti-NKG2A.IL-15 increased the killing of HLA-E expressing K562 cells, only β.anti-NKG2A.IL-15 induced a significant increase compared to medium control and β.anti-NKG2A (p = 0.0183 and p = 0.0194, respectively) (Additional File 1: Fig. S3C). We did not observe any difference in the degranulation and IFN-γ expression of CD8^+^ T cells (data not shown). Overall, these results suggest that the administration of rhuIL-15 or blocking the interaction with HLA-E alone is not sufficient to increase the cytotoxic capacity of NK cells in a similar way than the NKG2A.IL-15 NaMiX, indicating that the cytolytic effects of NaMiX require the synergy of both mechanisms.

### NaMiX induce activation, degranulation, IFN-γ and CXCR5 expression in PBMCs from HIV-1 infected individuals

**Fig. 6:**
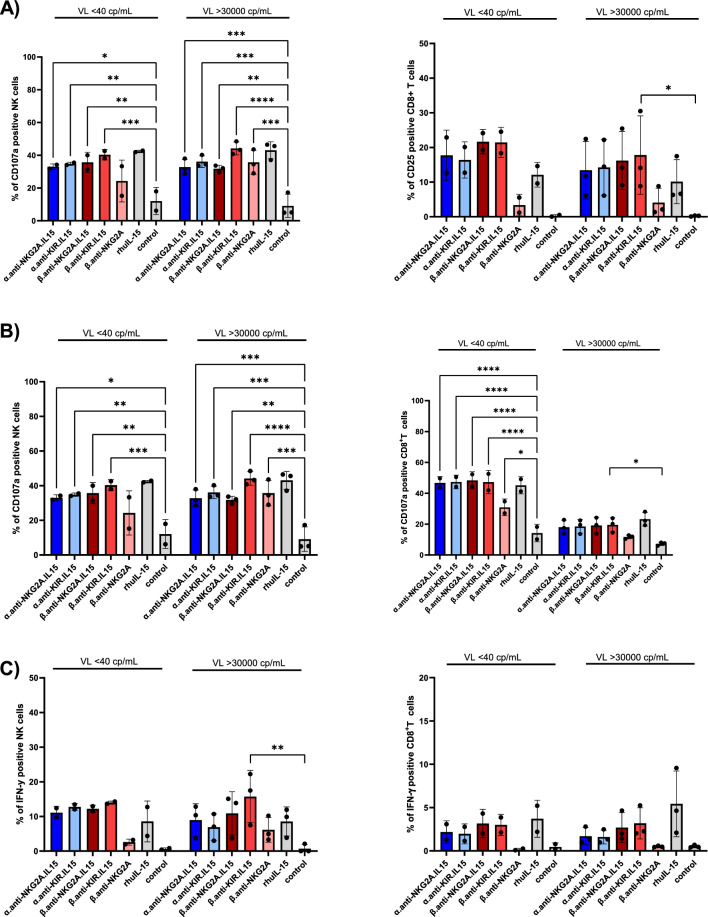

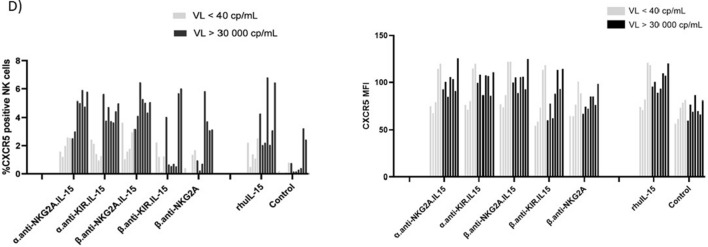
NaMiX increased activation, degranulation, IFN-γ and CXCR5 expression of PBMCs isolated from HIV-1 infected individuals. **A** Human PBMCs isolated from HIV-1 infected individuals treated with cART and displaying a viral load (VL) below 40 cp/mL (VL < 40 cp/mL) or not treated with cART and harbouring a VL above 30 000 cp/mL (VL > 30 000 cp/mL) were incubated for 48 h with the different molecules or not (control). Cells were further stained for extracellular markers to identify CD25 expression on CD3^−^CD56^+^CD16^+^ NK cells (left panel) and CD3^+^CD8^+^ T cells (right panel) using anti-CD3, CD8, CD14, CD16, CD19 and CD56 antibodies as compared to baseline expression (Control). **B** Human PBMCs isolated from the two groups of HIV-1 infected individuals were pre-incubated for 48 h with the different molecules and 5 h in presence of anti-CD107a (NK cells on left panel and CD8^+^ T cells on right panel). **C** Cells were also permeabilized and further stained for IFN-γ (NK cells on left panel and CD8^+^ T cells on right panel). The figure represents three independent experiments with different HIV-1 infected patients for each group. Data were expressed as the mean value ± SD. Statistical analysis was performed using a one-way ANOVA and post-hoc Tukey test (*p < 0.05, **p < 0.005, ***p < 0.001, ****p < 0.0001). **D)** Cells were further stained for extracellular markers to identify CXCR5 expression on CD3^−^CD56^+^CD16^+^. NK cells after 48 h in the presence of NaMiX as compared to baseline expression (control). Each bar represents CXCR5 expression or CXCR5 MFI of a single HIV-1 infected patient treated with cART for at least 2 years (grey bar, VL < 40 cp/mL) or before receiving cART (black bar, VL > 30 000 cp/mL)

The effects of NaMiX were further tested in samples from HIV-1 infected patients treated with cART for at least 2 years with an undetectable viral load (VL) or not treated with cART, and harbouring plasma VL below 40 copies/mL (cp/mL) or above 30 000 cp/mL, respectively. As shown in Fig. 6A, NK and CD8^+^ T cells were activated by NaMiX and upregulated the expression of IL-2 Rα, CD25, as an activation marker, similarly in the two groups of HIV-1 infected patients as compared to the β.anti-NKG2A control without IL-15 or rhIL-15. CD69 expression was similarly increased in all NaMiX-stimulated conditions in around 80 to 60% of NK cells or CD8^+^ T cells, respectively, for both groups of patients (data not shown). Regarding cytotoxicity, the different NaMix induced similarly increased expression of the degranulation marker CD107a on NK and CD8^+^ T (Fig. 6B) as well as IFN-γ in HIV-infected patient under cART or not (Fig. 6C). The presence of cytotoxic NK cells within BCF is a key component of SIV control in the nonpathogenic African green monkey model or SHIV viral replication in macaques. NK cells expressing the homing BCF receptor CXCR5 were recently shown to be inversely correlated with a superior control in these animal models but also with plasma VL in HIV-1 infected patients [13]. We therefore measured the expression of CXCR5 in PBMCs of HIV-1 infected patients stimulated for 48 h with the different NaMiX and observed that α.anti-NKG2A.IL-15 and β.anti-NKG2A.IL-15 were more potent to upregulate CXCR5 expression in NK cells (Fig. 6D) than their KIR counterparts or than β.anti-NKG2A in the different patients tested, and at a higher level (around threefold increase) in patients recently infected with HIV-1 with a VL above 30 000 copies/mL as compared to HIV-1 patients with chronic infection, and treated with cART for at least 2 years. We did not observe a significant increase in CXCR5 expression in CD4^+^ and CD8^+^ T cells (data not shown).

### NaMiX modulate HIV-1 replication in viral inhibition assays

We next examined the effect of NaMiX on the viral suppressive capacity of NK cells on CD4^+^ T cells from HIV-1 infected individuals who were under cART for at least 2 years. The heterogeneous basal infection level was broad among the patients, and the mean of change of p24 released by the CD4^+^ T cells after NaMiX stimulation was very large. Overall, we observed increased p24 positive CD4^+^ T cells after 2 days of incubation with NK cells stimulated with all molecules and rhuIL-15 (as shown for one representative donor in Additional File 1: Fig. S4A) suggesting a higher replication of the virus when stimulated with NaMiX at early time point. The latter could be due to the activation of NK and CD4^+^ T cells and the potential of IL-15 to reverse latency. After 5 days co-incubation with both α and β- IL-15-NaMiX or rhuIL-15 stimulated NK cells, a decrease of p24 positive CD4^+^ T cells was further measured. The β.anti-NKG2A control without IL-15 increased viral replication at 2 days co-incubation but did not decrease the intracellular p24 level after 5 days indicating that IL-15 alone is responsible for NK cell mediated viral inhibition. When looking at viral RNA in the supernatant, the levels were also slightly increased at 2 days co-incubation with stimulated NK cells compared to medium control (Additional File 1: Fig. S4B). In contrast, after 5 days of co-incubation with stimulated NK cells, NaMiX induced a decrease of HIV-1 RNA as compared to untreated NK cells, and this effect was similar to rhuIL-15 for α.anti-NKG2A.IL-15.

We further investigated whether NaMiX was indeed inducing or decreasing viral replication by activating human PBMCs (Red Cross Luxembourg) with NaMiX in the presence of latent HIV-1 infected ACH2 cells. As shown in Additional File 1: Fig. S4B, C, the activation marker CD25 was significantly increased by all forms of NaMiX in NK cells (p < 0.00005) and in CD4^+^ T cells (p < 0.05) but not with β.anti-NKG2A whereas the CD69 marker was induced by all molecules in NK cells (p < 0.0005) but not in CD4^+^ T cells. Next, we assessed the release of HIV-1 mRNA after 24 h of co-culture (Additional File 1: Fig. S4D) and observed an overall decrease of viral replication by all NaMiX as compared to non-stimulated ACH2 cells or IL-15 but not significant. Earlier time points showed low and similar mRNA levels among the different conditions, and the level of p24 expression measured by both flow cytometry and ELISA was also too low to observe a significant effect with ACH2 cells (data not shown). Taken together, these results suggest that NaMiX decrease viral replication of HIV-1 infected cells after cellular activation and killing of CD4^+^ T cells by stimulated NK cells.

### NSG tg-hu-IL-15 humanized mice develop more potent NK cells than NSG mice

We next wanted to evaluate whether NaMiX could induce cytotoxic NK cells in humanized mice infected with HIV-1 under cART. We have shown that humanized NOD.*Cg-Prkdc*^*scid*^*Il2rg*^*tm1Wjl*^/SzJ (NSG) mice infected with HIV-1 and treated with cART exhibit virological and immunological characteristics similar to HIV infection and HIV latency in humans [36]. However, due to a full IL-2Rγ knock out, human NK cell engraftment and maturation is very low in this mouse strain. Humanized NOD/Shi-*scid*-IL-2Rγ^null^ (NOG) or SIRPA Rag-/-IL2Rγ-/- (SRG) mice transgenic for human IL-15 (tg huIL-15) were developed to have more functional and more mature NK cells [41]. In order to characterize human NK cells, NSG and NSG tg-huIL-15 were humanized with CD34^+^ cells from human umbilical cord blood and sacrificed after 4 months to evaluate NK cell maturation and cytotoxicity. NSG tg-huIL-15 mice develop ten-times more human NK cells in blood (NSG mean: 2.3% ± 1.2%; NSG tg huIL-15: mean 22.03% ± 13.79%) and lung (NSG mean: 3.8% ± 1.4%; NSG tg huIL-15 mean: 36.9% ± 9.6%) and four-times more in spleen (NSG mean: 2.2% ± 0.63%; NSG tg huIL-15 mean: 8.9% ± 4.45%) compared to normal NSG mice (Additional File 1: Fig. S5A, B). Furthermore, NK cells from NSG tg-huIL-15 differentiate into CD56^dim^ and CD56^bright^ subpopulations in blood, lung and spleen (Additional File 1: Fig. S5C). However, there was only a small increase in overall KIR receptor expression on NK cells from blood (KIR2DL1/DS1 and KIR2DL2/DL3/DS2) and spleen (KIR2DL1/DS1) while NKG2A expression significantly increased in all organs of NSG tg-huIL-15 as compared to NSG mice (Additional File 1: Fig. S5D), reaching at least 60% of cells expressing NKG2A in the spleen and 80% in the blood, that was also observed in CD8^+^ T cells with a mean of 14% of cells expressing NKG2A in the spleen. To determine the cytotoxic activity of NK cells in both mouse strains, we incubated splenocytes with K562 cells and stained them for CD107a, intracellular IFN-γ and perforin expression. NK cells from humanized NSG tg-huIL-15 had a greater CD107a-reflected degranulation capacity against K562 cells, accompanied by a significantly higher perforin expression compared to NSG mice (p = 0.0186 and p = 0.0005, respectively) (Additional File 1: Fig. S5E). More importantly, splenocytes from NSG tg-huIL-15 were able to kill nearly 70% of target K562 cells (mean 68.75% ± 12.8%) while NSG splenocytes only killed 30% of target cells (mean 29.16% ± 23.61%) (Additional File 1: Fig. S5E). However, we observed no difference in IFN-γ expression in both mouse strains (Additional File 1: Fig. S5E). Overall, NSG tg-huIL-15 show a better NK cell engraftment after humanization, and a proper differentiation into fully cytotoxic and functional killer cells.

### The α.anti-NKG2A.IL-15 NaMiX increased NK cell cytotoxicity in vivo

**Fig. 7:**
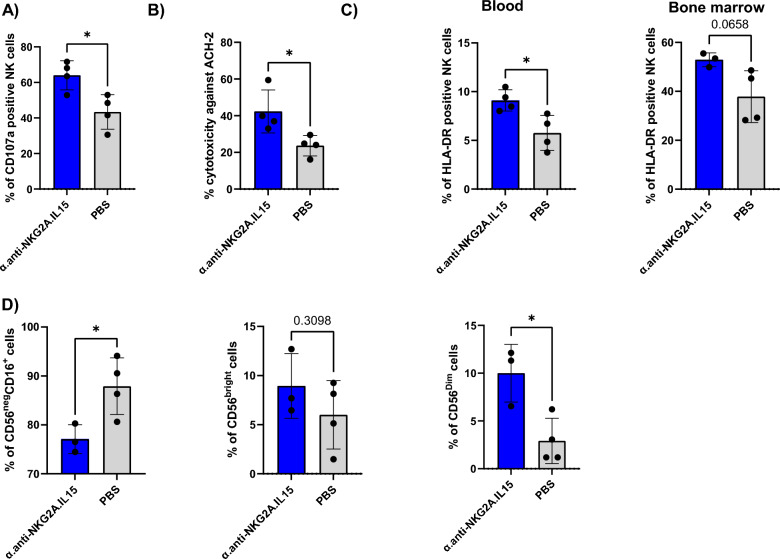
α.anti-NKG2A.IL-15 increased the cytotoxic activity of NK cells in infected humanized NSG tg huIL-15 mice treated with cART. Humanized NSG tg huIL-15 mice (n = 8) were infected with HIV-1 JRCSF for 4 weeks, treated with cART and injected with PBS (n = 4) or with the NaMiX engrafted with IL-15Rα/IL-15 using C4bpα and expressing the anti-NKG2A scFv (α.anti-NKG2A.IL-15, n = 4), ten and 3 days before treatment interruption. Mice were sacrificed and organs harvested 3 days after treatment interruption. **A** Splenocytes were incubated with ACH-2 cells for 5 h together with anti-CD107a and stained with anti-CD3, CD14, CD16, CD19 and CD56 antibodies to gate on CD3^−^CD56^+^CD16^+^ NK cells **B** ACH-2 cells were gated and evaluated for Live/Dead staining. **C** Blood (left) and BM (right) cells were stained to gate on CD3^−^CD56^+^CD16^+^ NK cells and the activation marker HLA-DR. **D** Comparison of NK subpopulations CD56^neg^ (left), CD56^bright^ (middle) and CD56^dim^. in the BM. Data were expressed as the mean value ± SD.Statistical analysis was performed using unpaired student t test (*p < 0.05)

Since α.anti-NKG2A.IL-15 was one of the lead NaMiX molecules to enhance NK cell degranulation and cytotoxicity against target cells in vitro, and given the high NKG2A expression of NK cells in all tissues in humanized NSG tg-huIL-15 mice (Fig. 7), we finally evaluated its efficacy in vivo in an HIV-1 latency model. Sixteen humanized mice were infected with HIV-1 for 4 weeks, treated with cART for 6 weeks and injected with the molecule or PBS (8 mice per group), ten and 3 days before treatment interruption, based on a dose previously used for the superagonist ALT-803 [19]. Four mice per group were sacrificed 3 days after treatment interruption to harvest cells from blood and spleen for phenotyping, cytotoxic activity and degranulation analysis against ACH-2 cells. NK cells from the spleen of mice treated with the lead NaMiX had a stronger CD107a expression against ACH-2 cells than PBS treated mice (64 ± 8.2% vs. 43.37 ± 9.7%) (p = 0.0176) (Fig. 7A). In addition, splenocytes from mice treated with α.anti-NKG2A.IL-15 killed more ACH-2 cells (42.38 ± 11.76%) as compared to NK cells from non-treated mice (23.69 ± 5.5%, p = 0.0283) (Fig. 7B). We also observed a slight but significant increase in the expression of the cell activation marker HLA-DR on blood NK cells from mice treated with NaMiX (mean 9.1 ± 1.09%) as compared to the PBS control group (mean 5.76 ± 1.79%) (p = 0.0465) (Fig. 7C). Even if not significant in the BM, the same tendency was observed (p = 0.0658). We additionally noticed a lower exhausted CD56^neg^CD16^+^ subpopulation in the BM of α.anti-NKG2A.IL-15 treated mice (mean 77 ± 2.93%) as compared to control mice (87.9 ± 5.8%) (p = 0.0332), which was compensated by a higher functional CD56^dim^ subpopulation (10 ± 3.0% vs. 2.9 ± 2.4%, p = 0.0173) (Fig. 7D). Interestingly, we did not see a difference in IFN-γ expression on NK cells nor a difference in CD8^+^ T cell activity between the two groups (data not shown).

**Fig. 8:**
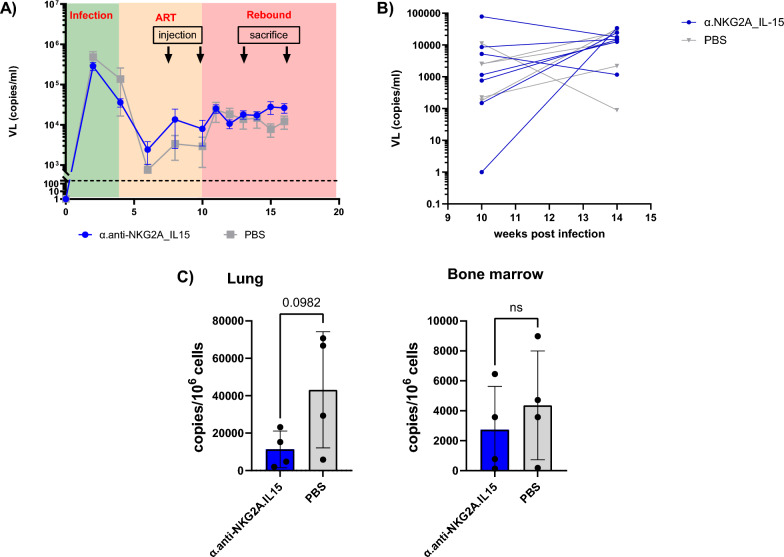
α.anti-NKG2A.IL-15 decreased HIV-1 reservoir in infected humanized NSG tg huIL-15 mice treated with cART. Humanized NSG tg huIL-15 mice (n = 8) were infected with JRCSF for 4 weeks, treated with cART and injected with PBS (n = 4) or with the NaMiX engrafted with IL-15Rα/IL-15 using C4bpα and expressing the anti-NKG2A scFv (α.anti-NKG2A.IL-15, n = 4), ten and 3 days before treatment interruption. Mice were sacrificed and organs harvested 3 days and 7 days after treatment initiation and the last injection of NaMiX (14 weeks after HIV-1 infection). **A** Viral load (VL) was measured every week or 2nd week. Left panel represents mean VL of the PBS and NaMiX-treated group. **B** Representation of viral rebound variation between treatment interruption and week 14 for each mouse in the right panel. **C** Total HIV-1 DNA of human CD45^+^. T cells in the lung (left panels) and in the bone marrow (BM, right panel) 3 days after the second injection. Data were expressed as the mean value ± SD. Statistical analysis was performed using unpaired student t test (*p < 0.05)

After cART treatment interruption, we monitored plasma VL over several weeks on eight additional mice (four in each group). In this first preliminary experiment, cART treatment did not decrease VL to an undetectable level for all mice in both groups (Fig. 8A) as it was previously observed in NSG mice [36], preventing the assessment of a viral rebound in an HIV-1 latency model. We nevertheless interrupted cART in these mice to see whether we could establish a general trend on VL. The PBS treated mice showed a heterogeneous VL distribution over time after cART interruption. In contrast, treatment with NaMiX result in a homogeneous increase in VL in all mice, which might indicate a potential effect of the molecule on viral replication (Fig. 8A) that might be due to latency reversal. In addition, when looking at the overall VL change from the time of cART interruption (week 10) to the end of the experiment (week 14), VL variation was higher in the PBS control group compared to the α.anti-NKG2A.IL-15 treated group (Fig. 8B).

To investigate the role of α.anti-NKG2A.IL-15 as a LRA in vivo*,* we measured total HIV-1 DNA in human CD45^+^ cells in BM and lung 3 days after the last injection of NaMiX and cART interruption in 4 mice from each group. Although no statistical significance was reached, which is most likely due to the low number of mice and the variability of VL in the control group, we observed a trend towards a decrease in total HIV-1 DNA in the lungs of mice treated with the NKG2A NaMiX compared to PBS (Fig. 8C) but not in the BM where immature NK cells reside. These preliminary data indicate that NK cells were stimulated by NaMiX and that NaMiX could have acted as a LRA in these mice. The dose and the time or duration of NaMiX delivery need now to be further optimized to evaluate its significant effect on viral rebound and to confirm its potential effect as LRA.

## Discussion

The present work demonstrates that targeted multimerisation of IL-15 on NK cells by the NKG2A receptor is a promising approach to enhance its stimulatory functions, in particular on NK cells isolated from HIV-1-infected patients. NaMiX increased cytotoxicity and killing activity of NK cells against HIV-1 infected cells in vitro and in vivo as well as against cancer cells in vitro. Overall, dimerization or heptamerization of the IL-15/IL15Rα and scFvs provided similar potency, especially for killing. Furthermore, some preliminary data suggest that NaMiX can provide additional benefits as a LRA to diminish the size of the viral reservoirs and increase the expression of CXCR5 in NK cells from HIV-1 infected patients.

The early establishment of a latent reservoir remains the key challenge for HIV-1 cure. The “shock and kill” therapy relies on the activation of latent reservoir cells by LRA followed by the recognition and elimination of cells harboring the reactivated virus by NK cells and cytotoxic T lymphocytes (CTL). Purging the entire reservoir without toxicity has proven to be challenging [42], [43]. New, safer, more specific and potent LRA have been developed, such as HDACi and HMTi [44–46], NF-кB modulators [47] and TLR agonists [48–50]. Yet, when used alone, none of these LRA was able to sufficiently decrease the reservoir size nor to delay viral rebound, indicating a deficiency in the “killing” of the reactivated cells. The functions and frequencies of cytotoxic NK and CD8^+^ T cells are highly affected as disease progresses towards a chronic phase, which makes them probably unable to clear the latent reservoir after viral reactivation [12]. Silencing the reservoir to avoid viral rebound can be achieved by targeted therapeutic vaccines promoting a sustained host immune response against HIV-1. The first vaccine trials aimed at priming CTLs against HIV-1 proteins [51, 52] or using autologous DCs pulsed with inactive HIV-1 [53, 54] or electroporated with mRNA [55] have not demonstrated sustained viral control. Furthermore, it was shown that vaccination with autologous DCs resulted in profound changes in NK cell phenotype and function, whereas expression of NKG2A and NKG2C remained stable during the course of vaccination [56]. Cocktails of broadly neutralizing antibodies (bNAbs) are currently the most promising approach to decrease VL to an undetectable level in infected humanized mice and rhesus macaques [57] or delay time to viral rebound in human [58]. Importantly, bNAbs seem to reduce viral reservoirs by stimulating the CTL response [57, 59], and combining latency reversal with bNAb treatment significantly prevented viral rebound after ATI for more than 6 months in half of the infected-treated macaques [60].

NK cells play a key role in the control of viral infection and they are now considered to support antiviral and HIV-1 cure strategies [4, 5, 61]. Immunotherapies priming NK cells against tumor cells by immune checkpoint inhibition or bispecific and trispecific killer cell engager (BiKEs and TriKEs) just underwent first phase clinical trials [62, 63]. In the non-pathogenic model of African green Monkey, a natural host of SIV, the terminally differentiated NKG2A^low^CD16^+^ phenotype is expanded, while there is accumulation of intermediary differentiated NKG2A^high^CD16^+^ NK cells in the pathogenic macaque model [64]. We propose here an immunoconjugate combining scFvs of the immune checkpoint inhibitors anti-NKG2A or anti-KIR2DL with the complex IL-15/IL15Rα, forming dimers or heptamers. At first, we demonstrated that multimerizing IL-15 at the surface of cells by targeting NKG2A significantly increased phosphorylation of STAT5 in NK and CD8^+^ T cells compared to IL-15 alone or by targeting KIR2DL. Signal transduction of IL-2Rγ_C_ chain stimulation can either occur through JAK1/3 mediated STAT5 phosphorylation [39] or PI-3-Akt-mTOR mediated phosphorylation of the ribosomal protein S6 [65], inducing survival or proliferation, respectively. Soluble IL-15/IL-15Rα preferentially activates the STAT5 phosphorylation pathway whereas trans-presentation by DCs or high levels of IL-15 activates S6 phosphorylation [66, 67]. IL-15 has long been known to enhance survival and expansion of exhausted NK and HIV-1 specific CD8^+^ T cells from HIV-positive individuals. In contrast, NaMiX did not stimulate the expression of the proliferation marker Ki-67 in PBMCs as compared to rhuIL-15 suggesting that the targeted delivery of IL-15 on NK cells using NK receptors may induce only cytotoxic activity of both NK and CD8^+^ T cells and not expansion. This feature could be of interest to avoid exhaustion of cells as described previously with continuous treatment of NK cells with rhuIL-15 resulting in decreased viability, diminished signaling, and decreased function [68].

We further showed that multimerizing IL-15 to NKG2A targeting increased degranulation and IFN-γ expression of NK cells against resistant cancer cells expressing multiple HLAs [69], while KIR2DL targeting did not show a significant effect probably due to reduced receptor expression. The expression of certain NK cell receptors, such as NKG2A and to a lower extent KIR2DL2/3, can be triggered on CD8^+^ T lymphocytes by T cell receptor (TCR) or IL-15 stimulation after a few cell cycles [70]. NKG2A blockade in combination with PD-1 blocking antibodies (monalizumab and durvalumab, respectively) was only pertinent when NK and CD8^+^ T cells were pre-stimulated with IL-15 for 9 days, which increased NKG2A and PD-1 expression [33]. Andre et al*.* further reported that monalizumab could restore CD107a expression on NKG2A^+^ NK cells against HLA-E positive K562 cells to a similar level seen with wild type K562 cells only when NK cells were pre-stimulated with IL-2 for 7 days. We noticed a significant increase in CD107a and IFN-γ expression on NK cells against HLA-E expressing K562 when incubated in vitro with α.anti-NKG2A.IL-15 or β.anti-NKG2A.IL-15 as soon as after 24 h of stimulation (data not shown). In our study however, CD8^+^ T cells stimulated with NaMiX did not increase IFN-γ expression when incubated with Raji or HLA-E expressing K562 cells, in agreement with data observed on CD8^+^ T cells primed with flu peptides and stimulated with rhuIL-15 and treated with monalizumab and durvalumab [33]. Similarly, Ohkawa et al. showed that cytokine-stimulated CD8^+^ T cells and NK cells, with increased IFN-γ secretion by NK cells, were unable to kill Raji cells [71]. Although blocking NKG2A restored the ability of NK cells to kill Raji cells in their study, we did not observe a significant effect of our β.anti-NKG2A control molecule on the degranulation or cytotoxicity of NK cells and CD8^+^ T cells against Raji targets. We further showed that anti-NKG2A.IL-15 NaMiX combining NKG2A blockade with IL-15 stimulation significantly increased cytotoxicity against HLA-E expressing K562 cells suggesting that NaMiX requires both functions to efficiently stimulate NK cells for killing, and that both mechanisms could synergize in vivo. We cannot exclude that our anti-KIR2DL scFv can reproduce immune checkpoint blockade on killing Raji cells when using the α or β forms of NaMiX since we were not able to produce the adequate controls without IL-15. Of note, all NaMiX increased degranulation and IFN-γ expression on NK cells and CD8^+^ T cells when exposed to HIV-1 positive ACH-2 cells. In contrast to Raji, HIV-1 positive cells activate the TCR signaling pathway on CD8^+^ T cells, which is the major driver of IFN-γ secretion [72]. Furthermore, control β.anti-NKG2A without IL-15 stimulated degranulation of NK cells with additional IFN-γ expression on CD8^+^ T cells as well, indicating that blocking the receptor in the context of viral infection might be more effective than in the context of cancer therapy. We did not observe significant differences between the α and β forms for both NKGAA and KIR NaMiX suggesting that targeted dimerization of IL15/IL15Rα complex is sufficiently potent as compared to targeted heptamerisation. We also showed that all NaMiX increased the killing capacity of PBMCs against ACH-2 cells to a similar level reported with 100 nM of the IL-15 superagonist ALT-803 [73]. With an estimated molecular weight of 200KDa, this represents 20 μg/ml of the superagonist, while we use 3 μg/ml of NaMiX. The killing seemed to be independent of ADCC, since addition of HIV-1 positive serum did not increase NaMiX cytotoxicity. However, ACH-2 cells do not express CD4, and antibodies from HIV-1 positive individuals required for ADCC preferentially target the CD4-gp120 bound epitope [74]. Lee et al*.* showed that ADCC was not induced by NK cells against ACH-2 cells, even when incubated with CD4 mimicking compounds [40]. Nevertheless, gp120 coated CEM.NKr-CCR5 target cells or primary T cells from HIV-1 positive individuals were able to stimulate ADCC [75]. Therefore, the effect of NaMiX on ADCC must be further investigated in additional studies.

Using the viral inhibition assay, we observed an increase in p24 levels and viral RNA after 2 days incubation with stimulated NK cells. This observation indicates a potential latency reversal effect of the molecules on chronically infected donors in accordance with results obtained in vitro with IL-15 and the superagonist ALT-803 [27] as well as in vivo in the phase I of ALT-803 [76]. Similarly, we observed a control of the viral replication when NK cells were pre-treated with NaMiX after 5 days of co-incubation with infected CD4^+^ T cells, strongly suggesting that NK cells were primed for killing infected cells. Furthermore, we observed a similar reduction in intracellular p24 levels when NK cells were stimulated with the IL-15 NaMiX and incubated for 5 days with infected CD4^+^ T cells than Garrido et al. showed with 25 ng/ml of rhuIL-15 after 7 days of co-incubation [3]. These authors demonstrated that IL-15 stimulated NK cells were able to detect and clear HIV-1 producing cells after latency reversal, forging our theory that NaMiX-stimulated NK cells could also clear reactivated CD4^+^ T cells.

Lymph node follicles serve as the primary site for cellular HIV-1 reservoirs due to the exclusion of immune cytolytic antiviral effector cells in BCF. Recent evidence raised from both animal models and HIV-1 infected patients have highlighted that CXCR5^+^ NK cells are promising effectors for HIV-1 immune control [13, 77]. We show here that NaMiX expressing IL-15 and targeting NKG2A upregulate in vitro the expression of CXCR5 in NK cells. In chronic SHIV infection, combining IL-12 and IL-15/IL-15Rα cytokine treatment potentiates both follicular homing and effector functions of NK cells [77]. The frequency of CXCR5^+^ follicular NK cells correlated with lower plasma VL in these animals and lower viremia in lymph nodes from HIV-1 infected individuals [13]. Although follicular cytotoxic T cells (Tfcs) expressing CXCR5 were identified in lymph node BCF during chronic SIV/HIV-1 infection, Tfcs do not express CXCR5, have weak cytotoxicity, and appeared to be non-HIV-1 specific [13]. In this regard, we did not observe an increase of CXCR5 in CD8^+^ T cells, but only in NK cells. In agreement, subcutaneous administration of ALT-803 in SHIV-infected rhesus macaques activates and mobilizes NK cells from the peripheral blood to lymph node B cell follicles [28], but higher CXCR5 expression could not be detected 3 weeks after the last dose after increased trafficking into BCF, probably because of a temporal regulation of CXCR5 expression.

We finally evaluated one of the lead molecules, α.anti-NKG2A.IL-15 NaMiX, in humanized NSG tg huIL-15 mice. These mice exhibited improved human NK cells reconstitution in lymphoid and non-lymphoid tissues (around 37% in the lung, Additional File 1: Fig. S5), as compared to NSG mice, with high degranulation, cytotoxicity and IFN-γ production against K562 cells ex vivo*.* These mice are therefore well adapted to study NK cell responses during HIV-1 infection as it was recently described for MISTRG-6-15 mice [78]. Furthermore, NK cells differentiate into functional CD56^bright^ and CD56^dim^ subpopulations in blood, lung and spleen. After 4 weeks of HIV-1 infection and 6 weeks of cART, the percentage of functional NK cells from both subsets decreased and the cells were less cytotoxic against HIV-1 infected ACH2 cells. Treating humanized NSG tg huIL-15 mice infected with HIV-1 under cART with α.anti-NKG2A.IL-15 increased the number of functional NK cells, their activation, degranulation and cytotoxic activity. Using two injections of a single dose before cART interruption, we did observe a tendency to decreased total HIV-1 DNA in the lung but not in the BM containing immature NK cells. This could indicate a latency reversal effect of NaMiX in vivo and a subsequent eradication of these cells by activated NK cells. This observation is in agreement with the results obtained in the viral inhibition assays with primary HIV-1 infected cells: a first increase of HIV1-mRNA was observed in the supernatant at 3 days followed by a decrease at 5 days (Additional File 1: Fig. S4). A first activation of CD4^+^ T cells by NaMiX might enhance viral replication followed by their subsequent killing by activated NK cells, as observed in ACH2 cells co-cultured with PBMCs (Additional File 1: Fig. S4). These results are supported by previous studies performed with rhuIL-15 or the superagonist ALT-803 in different animal models and in humans [73, 76, 79, 80]. It was recently shown that NK cells directly suppress in vivo HIV-1 replication in humanized MISTRG-6-15 mice by using NK cell depletion [78]*.* Interestingly, treatment of humanized MISTRG-6-15 mice with the PGT121 Nab modified or not with GRLR mutation showed that antibody treatment diminishes VL and potentiates NK cell cytolytic activity in an Fc-independent manner. Taken together, all these data suggest that new strategies to enhance NK cell function can contribute to the clearance of the virus and can be beneficial to combine with other therapeutic strategies towards HIV-1 cure.

Our study has several limitations. The use of humanized mice with a limited available blood volume/number of human cells has restricted a deep investigation of NK cell function, proliferation and expression of CXCR5 in NK cells after NaMiX treatment. A more robust decrease in the size of the reservoir and the stabilization of the VL in humanized mice could have potentially be achieved by earlier treatment with the molecules, at the start of cART, when NK cells and CD8^+^ T cells are less exhausted and more responsive to cytokine stimulation, as shown by others. Indeed, Seay et al. demonstrated that the superagonist ALT-803 induced viral control up to 21 days in an acute infection model of humanized mice [73], which was mainly driven by NK cell activation. Importantly, we did not address the implication of CD8^+^ T cells in the control of efficient latency on the HIV-1 reservoirs in the current work as suggested by Cartwright et al*.* [81]. The IL-15 superagonist ALT-803 significantly reversed latency when CD8^+^ T cells were depleted in SIV infected macaques and HIV-1 infected humanized mice under cART [79, 80]. This result is of great importance for further investigations with NaMiX and could explain that ALT-803 did not reverse latency in ART-suppressed humans and SHIV-infected macaques. Ultimately, to optimize the potency of NaMiX in vivo, further pharmacokinetic and pharmacodynamic studies will be essential, such as half-life, administration route, administration dose and toxicity. We expect an increased half-life of NaMiX compared to rhuIL-15, mainly due to the size of the molecule, which should decrease renal elimination. Multiple studies have enlarged the cytokine to increase the t_1/2_ of IL-15 by combining it with its co-receptor IL-15Rα, by attachment to an Ig-Fc domain or by pegylation [82]. The administration route of IL-15 has also been proven important in clinical trials; the first phase clinical trial with ALT-803 has demonstrated that subcutaneous (SC) injection increased the longevity of the molecule compared to intravenous (IV) injection in humans [83]. SC injection also significantly increased NK and CD8^+^ T cell count, maturation and activation in patients with hematologic malignancies that relapsed after allogenic hematopoietic cell transplantation.

## Conclusions

We demonstrated in the current work that multimerizing IL-15 at the surface of NK and CD8^+^ T cells increases the function and killing activity of both cell types in vitro. We showed preliminary data on the efficacy of NaMiX on NK cells in humanized mice. Our results are promising and open doors to new strategies for HIV cure by harnessing NK cells. NaMiX can be now evaluated for efficacy and toxicity in pre-clinical animal studies to optimize dose and mode of delivery.

### Supplementary Information

**Additional file 1: Fig. S1.** NaMiX bind to their respective receptors on NK cells. **A** The molecules containing the anti-KIR scFv were incubated for 30 min with HEK293F cells expressing KIR2DL1 (left), KIR2DL2 (middle) and KIR2DL3 (right) and stained with anti-His and anti-IL-15 for flow cytometry analysis. **B** The molecules containing the anti-NKG2A scFv were incubated with the stable cell line NK-92MI expressing NKG2A and stained with anti-His and anti-IL-15 for flow cytometry analysis. **C** PBMCs from different donors were stained for extracellular markers including KIR2DL1/DS1, KIR2DL2/L3/DS2 and NKG2A to identify and phenotype CD3^-^CD56^+^CD16^+^ NK cells (left) and CD3^+^CD8^+^ T (right) cells using anti-CD3, CD8, CD14, CD16, CD19 and CD56 antibodies. **D** All NaMiX were incubated with human PBMCs and stained for NK cell markers, anti-His and anti-IL-15 for flow cytometry analysis. Data were expressed as the mean value ± SD. Fig. **A** and **B** represent three independent experiments with three different donors. Fig. **C** and **D** represent six and three independent experiments, respectively, with three and six different donors. NaMiX engrafted with IL-15Rα/IL-15 using C4bpα or C4bpβ and expressing the anti-NKG2A or the anti-KIR scFv are so called: α.anti-NKG2A.IL-15, β.anti-NKG2A.IL-15, α.anti-KIR.IL-15 and β.anti-KIR.IL-15, respectively while the control NaMiX without IL-15Rα/IL-15 expressing the anti-NKG2A scFv is termed β.anti-NKG2A and the control condition without any molecules as medium. Data were expressed as the mean value ± SD. Statistical analysis was performed using a one-way ANOVA and post-hoc Tukey test (*p < 0.05, **p < 0.005, ***p < 0.001, ****p < 0.0001). **Fig. S2. **NaMiX increased STAT5 phosphorylation in NK and CD8+ T cells. **A **PBMCs were incubated with the NaMiX molecules for 1, 10, 20 or 40 min, stained on ice to gate for live CD3^-^CD56^+^CD16^+^ NK (upper panel) and live CD3^+^CD8^+^ T cells (lower panel), permeabilized on ice and stained for intra-cellular pSTAT5 for flow cytometry analysis. The left panels represent time-dependent pSTAT5 phosphorylation of one representative donor. Right panel represents the data of three different healthy donors at 1 min incubation. NaMiX engrafted with IL-15Rα/IL-15 using C4bpα or C4bpβ and expressing the anti-NKG2A or the anti-KIR scFv are so called: α.anti-NKG2A.IL-15, β.anti-NKG2A.IL-15, α.anti-KIR.IL-15 and β.anti-KIR.IL-15, respectively while the control NaMiX without IL-15Rα/IL-15 expressing the anti-NKG2A scFv is termed β.anti-NKG2A, the recombinant human IL-15 as rhIL-15, and the control condition without any molecules as medium. Data were expressed as the mean value ± SD. Statistical analysis was performed using a one-way ANOVA and post-hoc Tukey test (***p < 0.001, ****p < 0.0001). **B** PBMCs were incubated with the indicated molecules for 1 minute, stained on ice to gate for CD3^-^CD56^+^CD16^+^ NK cells and CD3^+^CD8^+^ T cells using anti-CD3, CD8, CD14, CD16, CD19 and CD56 antibodies, permeabilized on ice and stained for intracellular pSTAT5 for imaging flow cytometry. **Fig. S3. **IL-15 stimulation and blocking of NKG2A-HLA-E interaction were required for NK cell activation by NKG2A NaMiX. **A** Human PBMCs were incubated for 5 h with the NKG2A NaMiX and HLA-E expressing K562 cells in the presence of anti-CD107a mAb. Natural Killer cells were further stained for extracellular markers to identify CD107a expression. **B** Cells were also permeabilized and further stained for IFN-γ. **C** Human PBMCs were incubated for 5 h with the NaMiX and HLA-E expressing K562 pre-stained with CellTrace Violet. Cells were further stained for Live/Dead and analyzed by flow cytometry. The Figure represents three independent experiments with 3 different donors. NaMiX engrafted with IL-15Rα/IL-15 using C4bpα or C4bpβ and expressing the anti-NKG2A scFv are so called α.anti-NKG2A.IL-15 and β.anti-NKG2A.IL-15, respectively while the control NaMiX without IL-15Rα/IL-15 expressing the anti-NKG2A scFv is termed β.anti-NKG2A and the control condition without any molecules as medium. Data were expressed as the mean value ± SD. Statistical analysis was performed using a one-way ANOVA and post-hoc Tukey test (*p < 0.05, **p < 0.005, ***p < 0.001, ****p < 0.0001). **Fig. S4. **NaMiX increased viral inhibition capacity of NK cells against HIV-1 CD4^+^ T cells. Autologous CD4^+^ T cells and NK cells were purified by microbeads positive and negative selection, respectively, from HIV-1 individuals under antiretroviral therapy. CD4^+^ T cells were superinfected by spinoculation with HIV III-B and incubated with stimulated NK cells for two or 5 days at an E:T of 1:1. **A** p24 intracellular staining of CD4^+^ T cells of one representative donor (left panel) and HIV-1 RNA in the supernatant measured by ddPCR of one representative donor (right panel). The experiment has been performed with three different patients showing the same trend but with high baseline variability among them. **B **PBMCs of three healthy donors were pre-incubated for 48 h with the different molecules and stimulated with ACH2 cells for 5 h. Cells were further stained for extracellular markers to identify CD25 and CD69 expression on CD3^-^CD56^+^CD16^+^ NK cells and CD3^+^CD4^+^ T cells using anti-CD3, CD4, CD14, CD16, CD19 and CD56 antibodies. The Figure represents three independent experiments with three different donors. Data were expressed as the mean value ± SD. Statistical analysis was performed using a one-way ANOVA and post-hoc Tukey test (*p < 0.05, **p < 0.005, ***p < 0.001, ****p < 0.0001). **C** Representative expression of CD25 and CD69 on CD4^+^ T and NK cells pre-activated by NaMiX for 48 h for one donor included in panel B. **D** HIV-1 mRNA release in the supernatant of ACH2 cells stimulated for 24 h by human PBMCs prestimulated by NaMiX for 48 h. Three independent experiments were performed with three different healthy donors. Data were expressed as the mean value ± SD. Statistical analysis was performed using a one-way ANOVA and post-hoc Tukey test. **Fig. S5. **Humanized NSG mice transgenic for human IL-15 develop functional NK cells compared to NSG mice. NSG mice (n=4) or NSG tg huIL-15 mice (n=4) were humanized with human CD34^+^ cells from umbilical cord blood. After 4 months, mice were sacrificed and organs harvested. Cells were stained to gate on CD3^-^CD56^+^CD16^+^ NK cells with anti-CD3, CD14, CD16, CD19 and CD56 antibodies. **A** Representation of the dot plot of NK cells from the spleen of NSG (left) or NSG tghuIL-15 (right). **B** Comparison of NK cells in blood, lung, bone marrow (BM) and spleen in NSG mice and NSG tg huIL-15 mice. **C** Comparison of NK subpopulations CD56^dim^ (left) and CD56^bright^ (right) in blood, lung, BM and spleen. **D** Cells from blood, lung, BM and spleen were stained for NK cell markers such as KIR2DL1/DS1 (left) KIR2DL2/3/DS2 (middle) and NKG2A (right). Data were expressed as the mean value ± SD. Statistical analysis was performed using unpaired Student t test. (**p<0.005, ***p<0.0005, ****p<0.00005). **E** NK cells from humanized NSG mice transgenic for human IL-15 are cytotoxic as compared to NSG mice. Splenocytes were incubated with K562 cells for 5 h together with anti-CD107a (left graph), and were further permeabilized and stained with anti-perforin and anti-IFN-γ and anti-CD3, CD14, CD16, CD19 and CD56 antibodies to gate on CD3^-^CD56^+^CD16^+^ NK cells. K562 cells were pre-stained with CellTrace Violet and incubated with splenocytes for 5 h and all cells were stained for Live/Dead to determine cytotoxicity induced by NK cells. Data were expressed as the mean value ± SD. Statistical analysis was done using unpaired Student t test. (*p<0.05, ***p<0.0005).

## Data Availability

All data generated or analysed during this study are included in this published article.

## Abbreviations

Ab: Antibody
bNAbs: Broadly neutralizing antibodies
ADCC: Antibody-dependent cellular cytotoxicity
APC: Antigen presenting cells
BCF: B cell follicles
cART: Combined antiretroviral therapy
C4bp: Complement binding protein 4
cp/mL: Copies/mL
CTLA-4: Cytotoxic T-lymphocyte associated protein 4
CTL: Cytototoxic T lymphocytes
CXCR5: C-X-C chemokine receptor type 5
ELISA: Enzyme-Linked Immunosorbent Assay
HIV: Human immunodeficiency virus
HLA-C: Human leukocyte antigen class I-C
HLA-E: Human leukocyte antigen α-chain-E
IL-15: Interleukin-15
KIR2DL: Killer cell immunoglobulin-like receptors 2DL
NaMiX: Natural Killer activating multimeric immunotherapeutic complexes
NSG: NOD scid gamma mouse/LtSz-scid/IL2Rγnull
NK: Natural killer
NKG2A: Naturel killer receptor G2A or killer cell lectin-like receptor subfamily C, member 1A
LRA: Latency reversing agents
PBMCs: Peripheral blood mononuclear cells
PD1: Programmed cell death 1
PD-L1: Programmed cell death ligand 1
VL: Viral load
