# Supplementary material for: Multimeric immunotherapeutic complexes activating natural killer cells towards HIV-1 cure
Source: J Transl Med. 2023 Nov 7;21:791. doi: 10.1186/s12967-023-04669-4 (PMC10631209; doi:10.1186/s12967-023-04669-4)
Supplement: Supplementary file 1 — Additional file 1: Fig. S1. NaMiX bind to their respective receptors on NK cells. A The molecules containing the anti-KIR scFv were incubated for 30 min with HEK293F cells expressing KIR2DL1 (left), KIR2DL2 (middle) and KIR2DL3 (right) and stained with anti-His and anti-IL-15 for flow cytometry analysis. B The molecules containing the anti-NKG2A scFv were incubated with the stable cell line NK-92MI expressing NKG2A and stained with anti-His and anti-IL-15 for flow cytometry analysis. C PBMCs from different donors were stained for extracellular markers including KIR2DL1/DS1, KIR2DL2/L3/DS2 and NKG2A to identify and phenotype CD3-CD56+CD16+ NK cells (left) and CD3+CD8+ T (right) cells using anti-CD3, CD8, CD14, CD16, CD19 and CD56 antibodies. D All NaMiX were incubated with human PBMCs and stained for NK cell markers, anti-His and anti-IL-15 for flow cytometry analysis. Data were expressed as the mean value ± SD. Fig. A and B represent three independent experiments with three different donors. Fig. C and D represent six and three independent experiments, respectively, with three and six different donors. NaMiX engrafted with IL-15Rα/IL-15 using C4bpα or C4bpβ and expressing the anti-NKG2A or the anti-KIR scFv are so called: α.anti-NKG2A.IL-15, β.anti-NKG2A.IL-15, α.anti-KIR.IL-15 and β.anti-KIR.IL-15, respectively while the control NaMiX without IL-15Rα/IL-15 expressing the anti-NKG2A scFv is termed β.anti-NKG2A and the control condition without any molecules as medium. Data were expressed as the mean value ± SD. Statistical analysis was performed using a one-way ANOVA and post-hoc Tukey test (*p < 0.05, **p < 0.005, ***p < 0.001, ****p < 0.0001). Fig. S2. NaMiX increased STAT5 phosphorylation in NK and CD8+ T cells. A PBMCs were incubated with the NaMiX molecules for 1, 10, 20 or 40 min, stained on ice to gate for live CD3-CD56+CD16+ NK (upper panel) and live CD3+CD8+ T cells (lower panel), permeabilized on ice and stained for intra-cellular pSTAT5 for flow cytometry [file 12967_2023_4669_MOESM1_ESM.docx]

**Additional files: Supplementary figures 1 to 5**

**
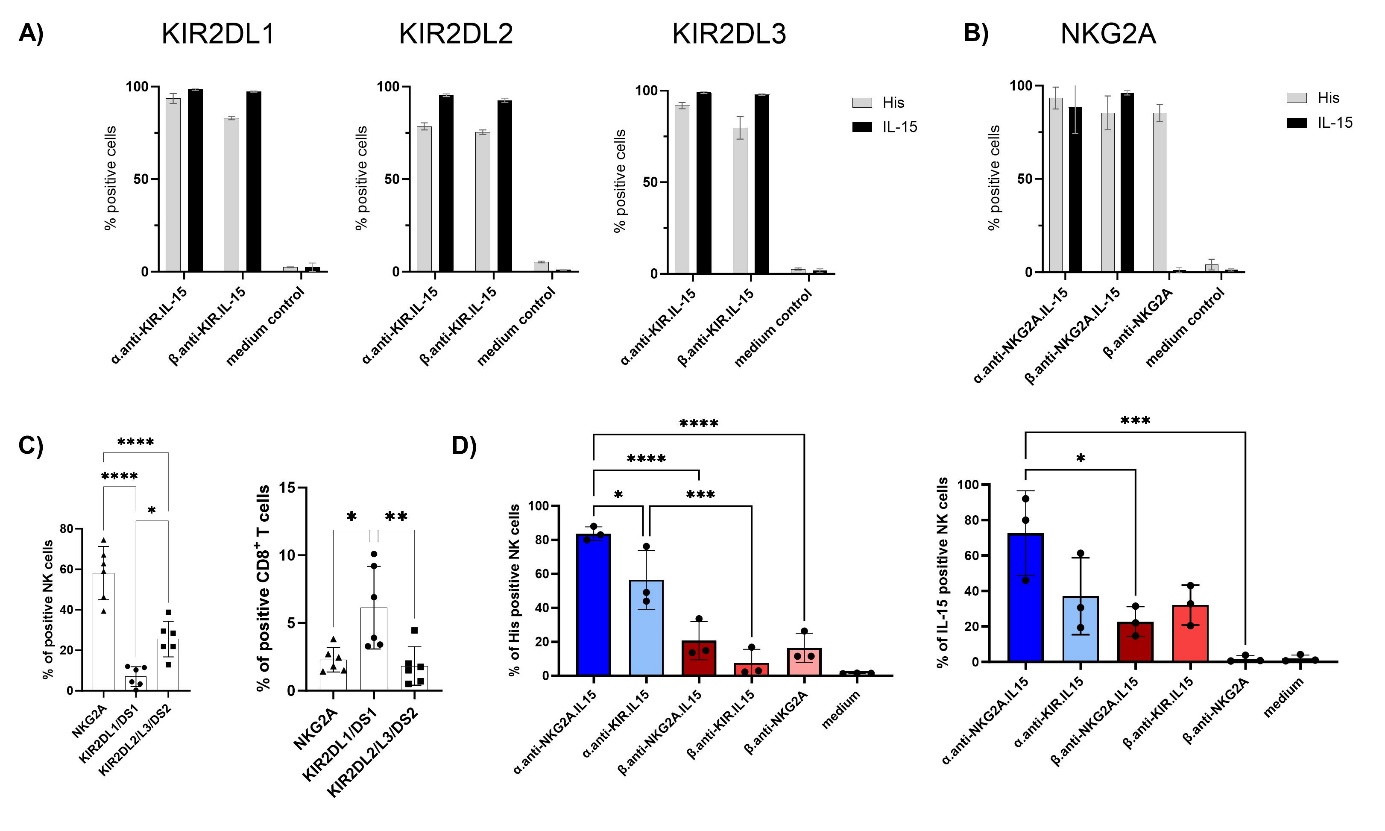
**

**Additional file 1: Fig. S1** NaMiX bind to their respective receptors on NK cells.


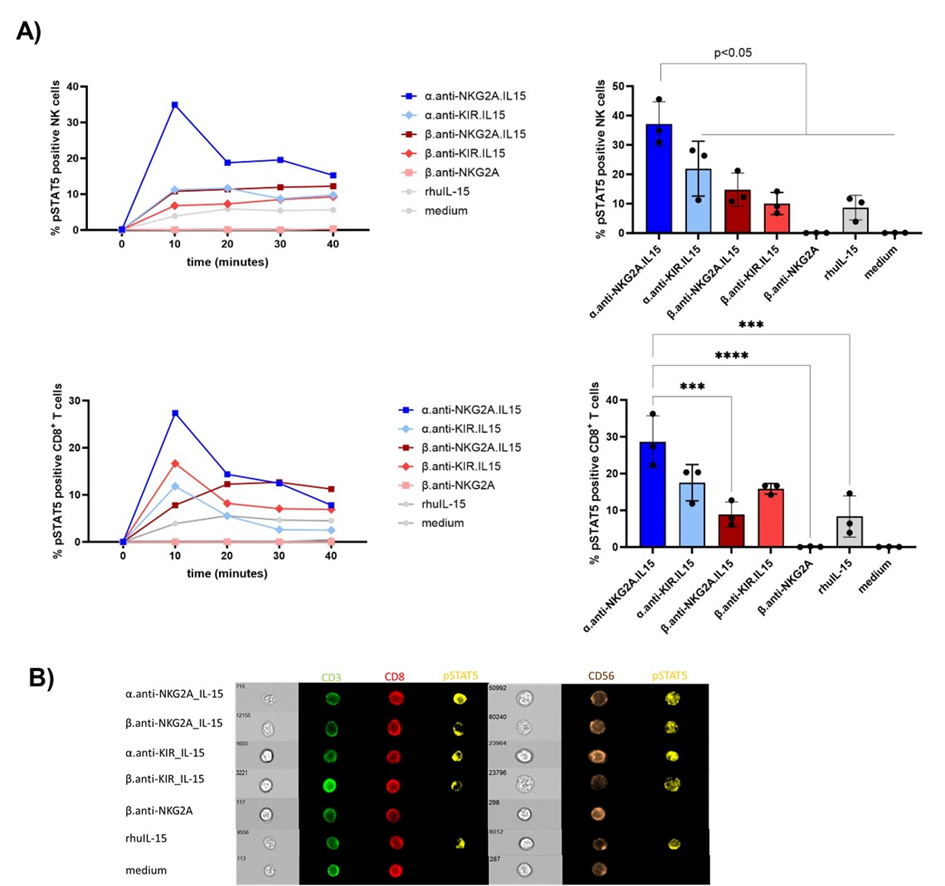


**Additional file 1: Fig. S2** NaMiX increased STAT5 phosphorylation in NK and CD8+ T cells.

**Additional file 1: Fig. S3** IL-15 stimulation and blocking of NKG2A-HLA-E interaction were required for NK cell activation by NKG2A NaMiX.

**Additional file 1: Fig. S4** NaMiX increased viral inhibition capacity of NK cells against HIV-1 CD4^+^ T cells.

**Additional file 1: Fig. S5** Humanized NSG mice transgenic for human IL-15 develop functional NK cells compared to NSG mice.
